# Roadmap for the multiscale coupling of biochemical and mechanical signals during development

**DOI:** 10.1088/1478-3975/abd0db

**Published:** 2021-04-14

**Authors:** Pierre-François Lenne, Edwin Munro, Idse Heemskerk, Aryeh Warmflash, Laura Bocanegra-Moreno, Kasumi Kishi, Anna Kicheva, Yuchen Long, Antoine Fruleux, Arezki Boudaoud, Timothy E Saunders, Paolo Caldarelli, Arthur Michaut, Jerome Gros, Yonit Maroudas-Sacks, Kinneret Keren, Edouard Hannezo, Zev J Gartner, Benjamin Stormo, Amy Gladfelter, Alan Rodrigues, Amy Shyer, Nicolas Minc, Jean-Léon Maître, Stefano Di Talia, Bassma Khamaisi, David Sprinzak, Sham Tlili

**Affiliations:** 1 Aix-Marseille University, CNRS, IBDM, Turing Center for Living Systems, Marseille, France; 2 Department of Molecular Genetics and Cell Biology, University of Chicago, Chicago, IL 60637, United States of America; 3 Department of Cell & Developmental Biology, University of Michigan Medical School, Ann Arbor, MI 48109, United States of America; 4 Department of Biosciences and Bioengineering, Rice University, Houston, TX, 77005, United States of America; 5 IST Austria, Am Campus 1, 3400 Klosterneuburg, Austria; 6 Reproduction et Dévelopement des Plantes, Université de Lyon, École normale supérieure de Lyon, Université Claude Bernard Lyon 1, INRAe, CNRS, 69364 Lyon Cedex 07, France; 7 LadHyX, CNRS, Ecole polytechnique, Institut Polytechnique de Paris, 91128 Palaiseau Cedex, France; 8 Mechanobiology Institute, National University of Singapore, 117411, Singapore; 9 Cellule Pasteur UPMC, Sorbonne Université, rue du Dr Roux, 75015 Paris, France; 10 Department of Developmental and Stem Cell Biology Institut Pasteur, 75724 Paris, Cedex 15, France; 11 CNRS UMR3738, 75015 Paris, France; 12 Department of Physics, Technion—Israel Institute of Technology, Haifa 32000, Israel; 13 Network Biology Research Laboratories and The Russell Berrie Nanotechnology Institute, Technion—Israel Institute of Technology, Haifa 32000, Israel; 14 Institute of Science and Technology Austria, Am Campus 1, 3400 Klosterneuburg, Austria; 15 Department of Pharmaceutical Chemistry, University of California, San Francisco, 600 16th St. Box 2280, San Francisco, CA 94158, United States of America; 16 Department of Biology, University of North Carolina—Chapel Hill, Chapel Hill, NC 27599 United States of America; 17 Laboratory of Morphogenesis, The Rockefeller University, 1230 York Avenue, New York, NY 10065, United States of America; 18 Institut Jacques Monod, Université de Paris, CNRS UMR7592, 15 rue Hélène Brion, 75205 Paris Cedex 13, France; 19 Institut Curie, PSL Research University, Sorbonne Université, CNRS UMR3215, INSERM U934, Paris, France; 20 Department of Cell Biology, Duke University Medical Center, Durham NC 27710, United States of America; 21 School of Neurobiology, Biochemistry and Biophysics, George S. Wise Faculty of Life Sciences, Tel Aviv University, Tel Aviv 6997801, Israel

**Keywords:** signalling, morphogenesis, embryogenesis

## Abstract

The way in which interactions between mechanics and biochemistry lead to the emergence of complex cell and tissue organization is an old question that has recently attracted renewed interest from biologists, physicists, mathematicians and computer scientists. Rapid advances in optical physics, microscopy and computational image analysis have greatly enhanced our ability to observe and quantify spatiotemporal patterns of signalling, force generation, deformation, and flow in living cells and tissues. Powerful new tools for genetic, biophysical and optogenetic manipulation are allowing us to perturb the underlying machinery that generates these patterns in increasingly sophisticated ways. Rapid advances in theory and computing have made it possible to construct predictive models that describe how cell and tissue organization and dynamics emerge from the local coupling of biochemistry and mechanics. Together, these advances have opened up a wealth of new opportunities to explore how mechanochemical patterning shapes organismal development. In this roadmap, we present a series of forward-looking case studies on mechanochemical patterning in development, written by scientists working at the interface between the physical and biological sciences, and covering a wide range of spatial and temporal scales, organisms, and modes of development. Together, these contributions highlight the many ways in which the dynamic coupling of mechanics and biochemistry shapes biological dynamics: from mechanoenzymes that sense force to tune their activity and motor output, to collectives of cells in tissues that flow and redistribute biochemical signals during development.

## 1. Introduction

The dynamic coupling of biochemistry and mechanics governs the organization of cells into tissues and organs during organismal development. Biochemical signals control force production and transmission within and between cells. These forces are resolved into subcellular deformation and flow, change of cell shape, cell movement, and ultimately collective movements and deformations of cell populations that drive the emergence of complex tissues and organs.

At the same time, force, deformation, and geometry feed back at multiple spatial and temporal scales to shape biochemical signals. Molecular motors and other mechanoenzymes sense force to tune enzymatic activity and motor output. Local deformations remodel cytoskeletal networks and adhesive contacts. Flows redistribute biochemical signals. Local changes in cell geometry promote the recruitment of curvature-sensing proteins. At the tissue level, dynamic changes in cellular shape and position, and local remodelling of cell-to-cell contacts, feed back to control signalling between cells and across tissues. The dynamic coupling of local biochemistry, force generation, and deformation governs the emergence of order at all scales: from adhesion complexes to self-organized structures such as the mitotic spindle and contractile actomyosin arrays, to the characteristic shapes of motile cells, to the emergence of complex functional structures such as muscles, branched vascular networks or the convoluted brain.

The way in which mechanochemical interactions lead to the emergence of complex cell and tissue organization is an old question. However, in recent years, this question has attracted a resurgence of interest from biologists, physicists, mathematicians and computer scientists. Rapid advances in optical physics, microscopy and computational image analysis have greatly enhanced our ability to observe, quantify and perturb spatiotemporal patterns of signalling, force generation, deformation, and flow in living cells and tissues. Rapid advances in theory and computing have made it possible to construct predictive models of how cell and tissue organization and dynamics emerge from the local coupling of biochemistry and mechanics. Physics provides a rich perspective on biological cells and tissues as active self-organized living matter, while biology provides a seemingly endless supply of examples in which different properties of living matter are encoded in molecular hardware.

In this roadmap, we bring together a series of forward-looking perspectives from scientists working at the interface of the physical and biological sciences. We highlight both recent successes and ongoing efforts to understand how the complex organization of cells and tissues emerges through mechanochemical coupling. We emphasize the need to understand the mechanisms that govern the production, propagation, degradation, and dissipation of biochemical and mechanical signals, and how these mechanisms determine the length and time scales on which signals are coupled and one type of signal can relay the other. By presenting case studies drawn from different systems and approached from different perspectives, we aim to highlight both the unique insights that can be obtained from studying particular systems and general themes that transcend the details of individual systems.

## 2. Dynamics of signalling in self-organized stem-cell assemblies

### 2.1. Status

Studying mammalian embryogenesis presents particular challenges, as *in utero* development precludes the observation and manipulation of live embryos. Recently, recapitulation of embryonic processes *in vitro* using pluripotent stem cells (PSCs) has emerged as a powerful alternative (reviewed in [1, 2]). Both two- and three-dimensional systems have been developed, and while the three-dimensional systems show a remarkable degree of patterning and morphogenesis, to date, a lack of reproducibility in size, shape, and pattern has largely hampered quantitative studies. In contrast, two-dimensional systems, while largely incapable of undergoing morphogenesis, are quantitatively reproducible, and have enabled investigators to begin to understand how self-organized paracrine signalling produces patterns in mammalian development.

The first two-dimensional patterning system mimicked gastrulation: the organization of germ layers in the mammalian embryo, starting from human PSCs (hPSCs) [1]. The patterning in this system is controlled by the same bone morphogenetic proteins (BMP), Wnt, fibroblast growth factor (FGF), and Nodal signals that pattern the embryo *in vivo*, and the germ layers are arranged in the same order, although they are placed adjacent to each other within the same plane rather than in a layered structure, as *in vivo*. Here, we focus on our expectations for continued discovery with these two-dimensional systems as well as the road towards quantitatively reproducible three-dimensional systems.

### 2.2. Current and future challenges

#### 2.2.1. Interpretation of dynamic signals

Recently, studies using micropatterned hPSCs showed that patterning does not result from the formation of static signalling gradients, but rather from dynamic waves of signals which propagate from the edge inwards specifying cell fates in their wake [1, 3]. Despite this progress, the precise relationship between signalling activity and fate remains unclear. The way in which the site of gastrulation, known as the primitive streak, is specified by Wnt and Nodal signalling remains an important outstanding question. It is clear that both signals are required, and that the cell fate is not a simple function of the concentration or duration of signalling, but the way in which cells integrate these signals into a fate decision is not understood. Similar questions pertain to how signals create patterns at other developmental stages. For example, a number of groups have recently developed two-dimensional hPSC models for ectodermal patterning into neural, neural crest, and future epidermis [4-6], however, the precise mapping between signalling dynamics and cell fates remains obscure here as well. In the future, quantitative simultaneous measurements of signalling and fate in live cells should provide insight into this question.

#### 2.2.2. Formation of signalling patterns

Complementary to the question of how extracellular signals are interpreted is the question of how these signals are generated and how the patterns of signalling activity that drive cell fate patterning are formed. To date, most studies have avoided this question by studying reporters of signalling activity directly without monitoring the ligands and inhibitors that modulate this activity. However, most models of patterning refer to diffusible extracellular ligands and their inhibitors (reviewed in [7]), and therefore directly observing these molecules is necessary for validating or disproving these models. This is a particularly challenging problem, as these molecules are secreted into the extracellular space and are often effective at very low concentrations. However, it is essential to observe the low endogenous levels since overexpression of these molecules may severely perturb their behaviour.

#### 2.2.3. Integration of chemical and physical signals

One emerging area is the intersection between mechanics and patterning. Mechanics may play an important role in specifying cell fates, as several of the signalling pathways involved in early development are known to be mechanosensitive, including Hippo and Wnt signalling. For example, recent work on mouse blastocysts directly links mechanical forces generated by myosin contractility to Hippo pathway activity and trophectoderm (TE) differentiation [8]. This suggests that a similar mechanism may control the extraembryonic differentiation of hPSCs. Little is known about the role of tissue mechanics in mammalian gastrulation or about its impact on early cell-fate decisions in hPSCs. This question can be separated into the role of substrate mechanics (including the extracellular matrix), and the role of intercellular forces. While soft substrates were found to enhance mesoderm differentiation (and in some settings extraembryonic differentiation), a broader understanding of how substrate properties affect self-organized patterning is missing [9]. Moreover, essentially nothing is known about the role of intercellular forces, which have not been measured or manipulated in embryoid systems.

### 2.3. Advances in science and technology to meet challenges

#### 2.3.1. Measuring endogenous ligand levels in live cells

Two-dimensional patterning systems may be ideal for studying the formation of signalling patterns, as they provide optimal imaging conditions. There is evidence that many ligand–receptor interactions occur basal to the cells [1], that is, between the cells and the coverslip, so it is possible that Total Internal Reflection fluorescence (TIRF) microscopy could be used to image these with single-molecule resolution. This would be an exciting opportunity, as it would represent the first live imaging of an endogenous extracellular morphogen gradient outside the limited studies that have been performed in *Caenorhabditis elegans* and Drosophila. Even if this fails, some insight may be indirectly gained. For example, recent studies using an inhibitor of Wnt secretion and live cell imaging of signalling activity have suggested that in the 2D gastrulation model, Wnt ligands are capable of long-range diffusion, while Nodal activity propagates primarily by autoactivation of Nodal production [3]. Other studies of Wnt have suggested that its range may be context dependent: it appears to act over a short range in the murine intestinal crypt, but over a long range in *C. elegans* embryos, so a system where the mechanisms of ligand dispersal could be dissected is of great interest.

#### 2.3.2. Reproducible stem-cell assemblies in 3D

Because of their high degree of reproducibility and ease of observation, two-dimensional stem-cell systems have enabled highly quantitative studies that have revealed surprising new features of pattern formation. However, no morphogenesis occurs in these systems, restricting their use to the study of isolated features of pattern formation. Moreover, 2D models are only available for a small number of developmental processes. 3D stem-cell systems display more realistic morphogenesis, and a wide range of 3D organoid systems are available [10]. However, detailed quantitative studies that aim to shed light on the mechanisms of self-organization in such systems are made very difficult by a lack of reproducibility: shape, size, and pattern show qualitative trends, but cannot be quantitatively compared between individual organoids/embryoids. For 3D models of the early embryo, this situation has significantly improved in the recent past [2]. Mouse stem cells have been made to form assemblies that closely resemble the early mouse embryo in shape and size. With microfluidic devices, hPSCs have also been made to form highly reproducible 3D structures resembling part of the early human embryo. If this technological development continues, it may soon be possible to gain deeper insight into how signalling orchestrates the interplay of patterning and morphogenesis. This will require not only precisely controlling 3D development, but also enabling high-resolution live-cell microscopy, which remains a challenge for these systems.

### 2.4. Concluding remarks

This is an exciting time for studying development with self-organizing stem-cell systems. Already, a number of models have been created that have led to significant insights into signalling dynamics and patterning. The development of technologies to measure extracellular ligands in space and time, to probe the mechanics of these structures, and to grow reproducible 3D cultures promises to expand this field to analysis of morphogenesis and its interplay with cell fates and tissue patterning.

## 3. Interplay between tissue growth, morphogen signalling, and pattern formation in development

### 3.1. Status

Tissue growth is essential for the transformation of a fertilized cell into a mature organism. To generate organs with reproducible shape and size, growth must be tightly coordinated with the specification of diverse cell fates and the signals that direct tissue patterning and morphogenesis. Yet, it is remarkable how little is known about this coordination.

During the past few decades, much progress has been made in unravelling how cell fates are specified. With the help of genetics, *in vitro* models of embryonic stem cell differentiation into diverse cell fates, and single-cell-sequencing technologies, a highly detailed picture has emerged of how cell fates are molecularly defined. Furthermore, our understanding of how morphogen signalling is interpreted to specify cell identities has advanced considerably since the ‘French flag’ problem was first posed by Wolpert. It is currently well established that patterns of cell-fate specification emerge from the dynamics of gene regulatory networks driven by signals that spread across tissues [11].

The greater understanding of the temporal dynamics of pattern formation has emphasized the fact that this process happens at the same time as tissues grow. However, tissue growth itself is poorly understood. While the cell-intrinsic regulation of the cell cycle has been extensively studied, it is unclear how global tissue-level control of tissue size is achieved.

Research into the Drosophila wing imaginal disc has contributed much to our knowledge about this question. This organ possesses an intrinsic size-control mechanism, which allows it to measure its absolute dimensions. Signalling by the morphogens Dpp and Wg is required for the correct growth of the wing disc, although the precise mechanism of how size regulation is achieved is still a matter of intense investigation [12]. While the existence of intrinsic size control mechanisms is not so well established in other systems, it is clear that organs have reproducible dimensions and shapes characteristic of their species. Furthermore, similarly to imaginal discs, the signalling molecules that specify cell identities also regulate cell-cycle progression and cell survival in many different organs and systems.

**Figure 1.**
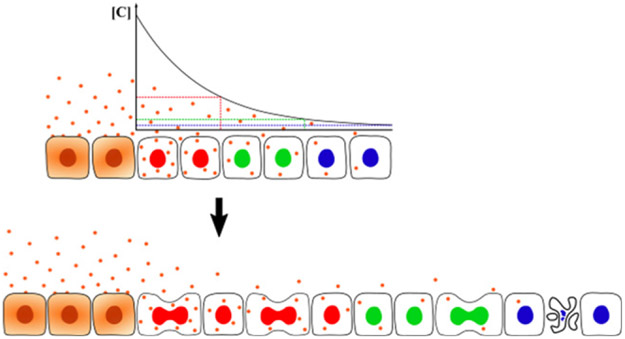
Illustration of a hypothetical morphogen-regulated developing tissue. Morphogen production by a restricted source (orange), spreading and degradation lead to the formation of a concentration gradient across the tissue. Target cells interpret this gradient and adopt red, green or blue cell identities. In this example, high morphogen concentrations promote cell proliferation and cell survival. As the tissue grows, morphogen levels in the expanding tissue are diluted by cell division, target cells move away from the source and the source itself also grows. The balance between these processes affects the final gradient shape, pattern and growth of the tissue.

Our current knowledge paints a picture in which multiple feedback loops exist between tissue growth, morphogen signalling and pattern formation (figure 1). On one hand, morphogens control both cell fates and tissue growth. On the other, signalling profiles and cell positions are modulated by tissue growth. Furthermore, pattern can locally alter the parameters of tissue growth. Further investigation of these feedbacks are necessary to gain an insight into how they work together to help achieve reproducible organ size and shape during development.

### 3.2. Current and future challenges

A major obstacle to advancing our understanding of the interplay between growth, signalling and pattern formation is our poor understanding of tissue growth itself. This is perhaps not surprising—growth control is multifaceted and occurs at multiple scales. Systemic and nutritional inputs act alongside tissue-level regulators such as morphogens and survival factors to regulate the dynamics of cell-intrinsic machineries. Although the way in which individual cells interpret tissue-level signalling to regulate their cell cycle progression is poorly understood, in many cases these signals have quantitative, rather than all-or-none, effects on tissue size. For example, the overexpression of Dpp results in enlarged wing discs, whereas hypomorphic mutants produce smaller wings [12]. Nevertheless, it is still debated whether morphogens act in an instructive or permissive manner to control growth. It is also unclear how size is sensed, how perturbations in size are corrected and how morphogens contribute to these processes.

Tissue-level regulators of growth converge downstream on the core molecular machineries driving cell cycle progression, cell death and cell growth. This can occur indirectly via the effects of morphogens on pattern formation. For instance, in the neural tube, progenitor identities are initially specified by morphogen signalling; subsequently, different progenitor types exit the cell cycle at distinct rates [13]. Signalling by extrinsic factors can also directly affect the core cell cycle and cell-survival regulators. What key components and interactions are regulated to alter the cell cycle speed, the probability to progress versus exit the cell cycle or undergo apoptosis and how cell type specificity of this regulation is achieved are open questions.

While we need a better understanding of how tissue growth is controlled, it is also necessary to consider that growth may affect its own tissue-level regulators in several ways. Signalling molecules can be diluted due to growth and advected away from their source of production, thereby altering the morphogen gradient shape. Moreover, tissue growth can change the positions of target cells with respect to the tissue boundaries and sources of morphogen production (figure 1). At the same time, growth of the sources of morphogen production alters the overall morphogen production rates over time. This contributes to temporal changes in signalling in individual cells. For instance, the posterior elongation of the vertebrate body axis over time leads to the displacement of cells away from FGF produced in the tailbud. The restricted time interval of FGF activity is key for correct pattern specification [14].

A central challenge that lies ahead is not only to advance our knowledge of the interactions between signalling, pattern and tissue growth, but also to understand the emergent properties of these interactions. Tissue growth could be viewed as a process that modifies itself via its relationship to signalling and pattern. This is likely to be fundamental for the self-organising properties of developmental systems [15]. Furthermore, in analogy to principles from control theory, these feedbacks could underlie the reproducibility of organ-size determination and patterning [16].

### 3.3. Advances in science and technology to meet challenges

Tissue growth and pattern formation are dynamic processes that elicit quantitative changes in tissue morphology and depend on multi-scale regulation. To capture these defining features, it has been of crucial importance to obtain high-resolution spatiotemporal quantitative data and use theoretical models to interpret and understand the underlying complexity.

In recent years, advances in microscopy and imaging assays have allowed *in vivo* observation and quantitative measurements of the dynamic cell and molecular behaviours that underlie tissue growth and pattern formation. For instance, light-sheet microscopy has allowed the visualisation of cellular morphodynamics in Drosophila, zebrafish and mouse embryos, as well as in organoids, over extended periods. Combined with real-time data processing to accommodate for significant embryo growth, this has recently allowed the imaging of large and complex specimens, such as the post-implantation mouse embryo up to organogenesis stages [16]. At the molecular level, assays based on fluorescence recovery after photobleaching (FRAP), fluorescence correlation spectroscopy (FCS), and photoactivation allow the monitoring of the nonsteady state kinetics of morphogens as they spread through tissues. Photoactivatable proteins fused to receptors in opto-genetic chimeras, as well as fluorophores with defined half-lives that constitute fluorescent timers, have provided novel methodologies for studying the dynamics of morphogen signalling and gradient formation [18, 19].

New developments in genetics and genome engineering have also been instrumental. These have permitted the tagging of endogenous ligands, as well as the precise manipulation of the components of signalling pathways. In addition, advanced mosaic cell-labelling techniques have allowed dynamic tracking of cell lineages as well as the properties of tissue growth in different species. For instance, analysis of the size and shape of clones of lineage-related cells labelled at defined developmental stages has provided information about the growth rate and anisotropy of tissue growth in the mouse neural tube [20] and heart [21]. This provides useful information for understanding the links between tissue growth and morphogenesis. Furthermore, the ability to track growth parameters simultaneously with cell lineage makes clonal analysis a promising approach for understanding how pattern formation and growth are coordinated during development.

The advances in the type, quality and resolution of experimental data demand new theoretical models to interpret these data. Dynamical systems and diffusion models that capture key biophysical properties and interactions have been useful for understanding specific processes, such as morphogen-gradient formation, gradient scaling with tissue size or the interpretation of morphogen signalling by downstream patterning regulatory networks. The development of models that integrate the knowledge of how these different processes interact, however, is still a challenge. Computational models in which cells are represented as discrete units interacting via mechanical forces are increasingly used to obtain a more realistic picture of the dynamic relationships between growth, signalling and morphogenesis during development. Such models are likely to become more elaborate as they develop into indispensable platforms for the synthesis and analysis of experimental data.

### 3.4. Concluding remarks

Recent technological advances are leading to substantial progress in understanding the mechanisms of morphogen signalling, pattern formation, and tissue growth. Establishing experimental and theoretical methods that would allow unraveling the complex interactions between these processes and achieve an understanding of the emergent properties of morphogen-driven systems is a key future challenge. The open questions discussed in this review are indicative of an exciting time to be working in the field of developmental biology, where there is still much to be discovered and understood.

## 4. The biophysical basis of robust plant morphogenesis

### 4.1. Status

**Figure 2.**
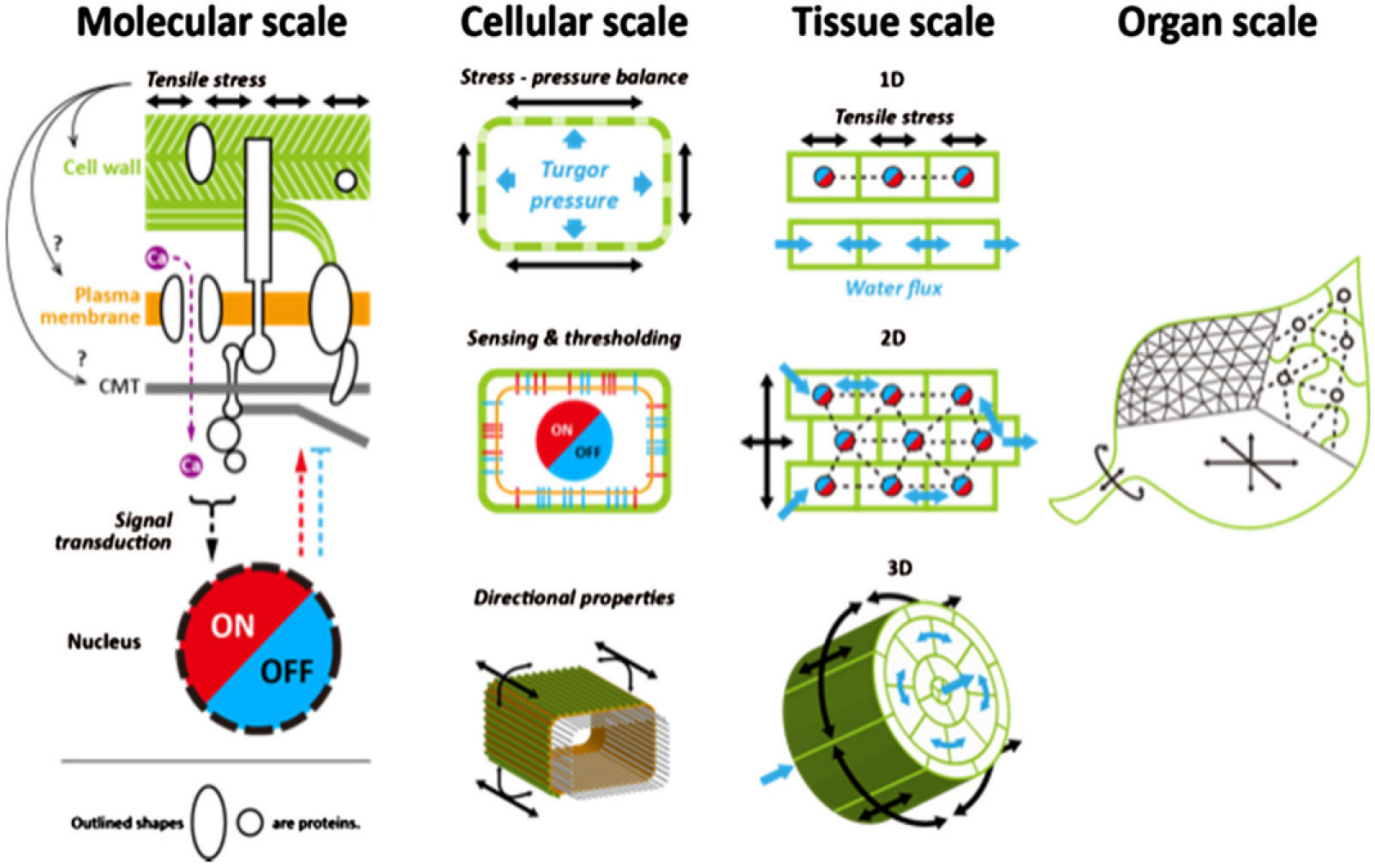
Plant morphogenesis is a multiscale and multilevel problem.

Morphogenesis, or the transformation of a developing organism to achieve a well-defined shape and size, has historically been envisioned as taking place through genetic programing. As such, the reproducibility of organ morphology observed in nature (e.g. the symmetry of the two hands of a human, the invariance of flowers within a plant) find its explanation in that every organ follows approximately the same programmed transformation. Nevertheless, a growing organism encounters multiple internal and external perturbations (mechanical, nutritional, pathological, etc.); therefore, a certain variability in final organ shape and size could be expected. The relatively low variability at organ level suggests that, besides following a preprogrammed transformation, organogenesis responds to perturbations and can limit the fluctuations in final organ morphology. Tissue mechanics has emerged as a key component in morphogenesis, both in making the link between gene expression and shape changes and as a buffering mechanism to achieve developmental robustness. We are, however, far from fully understanding the role of tissue mechanics in morphogenesis (figure 2).

Since their growth involves relatively simple physics and is slow enough to be easily observed, plants are ideal systems to investigate these issues. Plant-cell growth involves a balance between the turgor pressure (hydrostatic pressure caused by an actively maintained osmotic flow) and the tension in the encasing cell wall. On developmental timescales, part of the elastic tension in the cell wall is released by its expansion via remodelling and the delivery of new material. Mechanical concepts in plant development date back to the 19th century with, for instance, experiments demonstrating that the plant epidermis is generally under tension. Such concepts were somehow overlooked during the 20th century, with the notable exception of scientists such as Paul Green, until a revival prompted by recent studies (reviewed in [22]). The positioning of flower primordia (early floral buds) at the shoot tip was associated with changes in cell-wall composition and stiffness, hinting at a link between gene expression and tissue mechanics. The cytoskeleton of cortical microtubules (MTs) was found to respond to mechanical forces, contributing to the robustness of morphogenesis at the shoot apex. Similar processes were identified in the root, while new approaches have been developed or renewed to quantify plant mechanics at the cellular or tissue scales, including the pressure probe, nano-indentation [notably atomic force microscopy (AFM)], and extensometry (reviewed in [23]).

Many theoretical approaches were developed in parallel. While a class of models, kinematic models [24], tried to find the minimal local rules relating gene expression to growth patterns that reproduced the complex shapes observed in nature, other models tried to relate these shapes to tissue mechanical properties [22, 25]. From this biomechanical perspective, different questions arise depending on the scale considered. At a subcellular scale, models relate tissue elasto-plastic properties to the cell wall’s composition or assess the cell wall’s response to mechanical stress [22]. At a cellular scale, models try to relate cellular parameters, such as cell size, wall tension and turgor pressure which can, in turn, describe the upper organ scale. Cellular models such as vertex models reduce the complexity of cellular functions to the consideration of a few features and deduce the tissue behaviours at a macroscopic scale. Other models adopt a coarser viewpoint and describe tissues as continuous media. While coarsegrained models allow the depiction of more general tissue properties, cellular models can consider more subtle effects such as the coupling between hydraulics and cell mechanics. Altogether, these mechanical models investigate plant tissues’ mechanical responses and their impact on morphological robustness.

Here, we discuss new challenges in the mechanics of plant morphogenesis that stem from the parallel rise in quantitative experiments and theoretical models.

### 4.2. Current and future challenges

Biological complexity underlies many aspects of the challenges in understanding plant morphogenesis.

#### Plants as complex biomechanical systems.

The cell wall is a thin layer composed of many polysaccharides (cellulose, pectins, hemicellulose) and structural proteins with variable chemistry and composition, all of which are ultimately regulated by genes. The mechanical properties of the cell wall differ according to direction (in-plane, perpendicular to the plane) and may vary in space and in time, according to cell type and/or to cell status [26]. The relevance to growth of these properties is unclear, as they are measured at timescales much shorter than those of growth. Turgor pressure depends on the regulation of all cellular osmolytes (involving movement, active transport, and chemical reactions) and water movement in/out of the cell [27]. It is still unclear exactly how the cellular growth rate is determined, while there is a strong need to globally bridge the genetic, biochemical, and biomechanical statuses of cell wall, which are mostly considered separately.

#### Specificity of mechanosensing and mechanotransduction.

Whereas the molecular basis of mechanosensing (force-induced protein conformational changes) is generally recognized, collective behaviours of mechanosensitive proteins are less understood: how much signal, detected by different proteins, is required to initiate an active response? How does the MT cytoskeleton respond directionally to forces? Given the biological complexity, how is specificity ensured in mechanical signal sensing and transduction? For example, is stress, strain or a secondary signal sensed, and where [28]? How is the mechanically-stimulated Ca^2+^ signature, a common secondary messenger, perceived differentially from biotic stress and other signals [29]? Capturing the key determinants is crucial to understand specific responses to mechanical signals.

#### Mechanical variability at multiple scales.

Numerous studies have revealed that most, if not all, plant biomechanical properties are spatially variable [30]. If and how variability contributes positively to robustness is still an open question. What is the temporal variability of mechanical properties? What are the corresponding characteristic (correlation) times and lengths? How are the mechanical properties averaged over space and time and how does such averaging relate to morphological robustness? Finally, as heterogeneity in mechanical properties and/or in growth rate induces mechanical stress in the tissue, do mechanosensing and mechanotransduction contribute to robustness, and if so, how?

### 4.3. Advances in science and technology to meet challenges

Conceptual and technical advances in data acquisition, data analysis, physical modelling and simulation are required to meet the challenges mentioned above.

#### Multidimensional data acquisition.

Current biomechanical studies are largely limited to the surface of plant tissue (the epidermis and outer cell wall). So far, researchers have coped with this limitation by resorting to the theory that plant growth is controlled by the epidermis. Yet it is increasingly being realised that the inner tissues also contribute significantly to plant biomechanics. Increased detection distance and spatial resolution will promote the optical imaging of deeper tissues. The use of biosensors (e.g., sensors for cytoskeletal tension or for pressure), validated in the epidermis by direct biophysical measurements, will give mechanical information about deeper tissues. Meanwhile, higher temporal resolution demands automation with minimized invasiveness (e.g. reduced phototoxicity and effects of mechanical probing). All these will benefit from new methods and optimizations for optical/mechanical measurements, such as super-resolution optical microscopy, elastography (the optical inference of mechanical properties), or high-speed AFM [23, 31].

#### Data analysis and pattern recognition.

Compared to physical systems, the number of replicates in biophysical experiments is often technically limited, while biological variability complicates conclusions. Assessing 3D tissues, to multiply the quantity of data produced, will require the development of fully automated methods of data extraction. To establish correlations between biological data of increasing dimensions (geometry, fluorescent reporters, mechanics… at multiple scales), it is now common to use linear correlation analyses, mutual information, and dimensional reduction such as principal component analysis. Methods from information theory and machine learning could become even more powerful when they include constraints provided by physical laws.

#### Models and quantitative comparison with experiments.

Whereas models are increasingly qualitatively compared with experiments, the lack of quantitative comparisons leaves it unclear whether current theoretical frameworks are sufficient to describe morphogenesis in plants. This raises the issue of inferring model parameters from experiments, as performed for traditional physical systems. Inference could appear to be infeasible, given the complexity of some biophysical models and their large number of parameters. Nevertheless, classical approaches to dimensional reduction in continuum mechanics, such as homogenization (from discrete to continuous models) or approximations of thin sheets (for leaves) or rods (for stems), may simplify models and reduce the number of their parameters, facilitating model exploration and parametrization.

### 4.4. Concluding remarks

The time is ripe to further integrate experiment and theory and bring plant biophysics closer to the quantitative standard set by traditional physical systems. This will involve closing the cultural gap between biologists and physicists, and enabling research to combine molecular genetics, mechanical measurements, and modelling. We will thus make progress in a multiscale and multilevel understanding of the biophysics of plant morphogenesis.

## 5. Dynamics of morphogen gradient formation and signalling during development

### 5.1. Status

**Figure 3.**
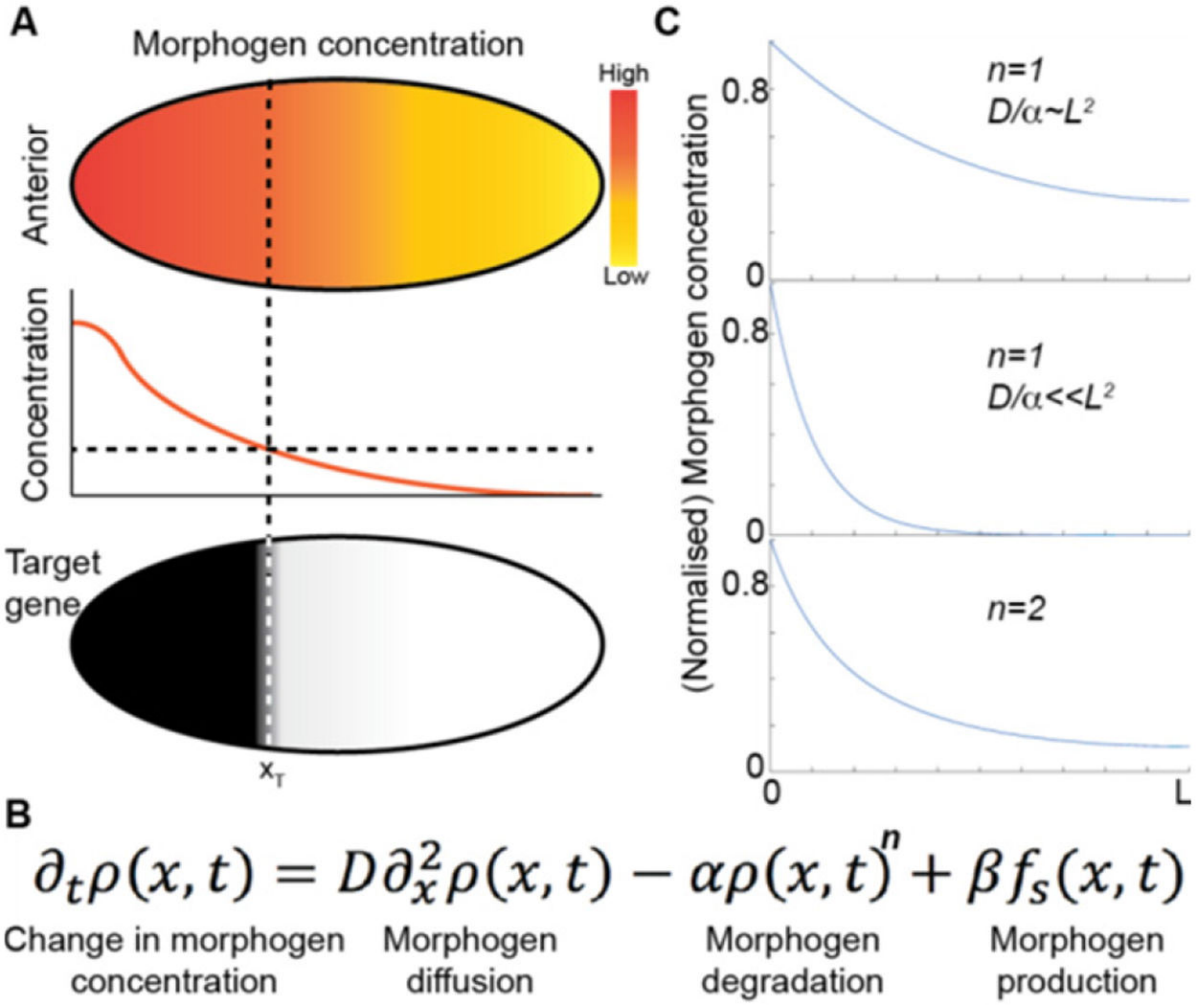
(A) (Top) morphogen gradients form spatially varying concentration gradients across the system. (Middle) at threshold concentrations (dashed line), different downstream gene responses occur (bottom), defining spatial boundaries in the system. Reproduced from [18] under the CC BY 4.0 license. (B) Reaction–diffusion model for morphogen gradient formation (morphogen concentration denoted by *ρ*). SDD model, *n* = 1. (C) Steady-state profiles for the model shown in (B) for differing degradation terms, *L* = system length.

Morphogens provide crucial spatial and temporal information to developing systems. They form concentration gradients across the system (which can be a single tissue or the whole embryo), and the gene response is dependent on the local morphogen concentration (figure 3(A)). The morphogen concept was proposed by Alan Turing in 1952 [32], yet the first experimentally identified morphogen, Bicoid in the early *Drosophila* embryo [33], was not found until the 1980s. Subsequently, various morphogens have been identified—e.g. FGF, Wingless (a member of Wnt family) and BMP—that play essential roles in many developmental processes, from genetic boundary specification to the regulation of growth. Defects in the morphogenetic programme result in a plethora of birth defects, which are often lethal. Hence, understanding how gradients form and how they are interpreted is essential in deciphering how complexity of form emerges during development.

A classic model of morphogen gradient formation is the source, diffusion, degradation (SDD) model [34] (figure 3(B)). A source of morphogen is localized to a specific spatial region of tissue. The morphogen then randomly diffuses through the tissue. Finally, protein degradation helps shape the gradient (figure 3(C)). The SDD model results in an exponentially-decaying morphogen concentration in a steady state; as commonly observed *in vivo* [34, 35]. Different forms for the degradation can result in power-law-like gradients (figure 3(C)). The mechanism of morphogen degradation (trapping) shapes the gradient and also determines the timescale within which the morphogen reaches its steady state [18].

Development is highly temporally coordinated; multiple events must occur in a specific order to ensure that development proceeds correctly. For example, during specification of the vertebrate neural tube, the temporal sequence of cell fate determination is highly dynamic, with temporally varying gene expression in cells at particular spatial positions [36]. However, in most developing systems there is still much to learn about how processes are coordinated both temporally and spatially to ensure robust morphogenesis.

### 5.2. Current and future challenges

#### 5.2.1. Dynamics of morphogen gradient formation

What are the dynamic processes that form morphogen gradients, and on what timescales do these processes act? The answer to these questions remains hotly debated for most morphogens. Bicoid is most likely to be the best-understood morphogen in terms of its dynamics [33], and it is qualitatively well described by the SDD model [18, 34]. Despite being accessible for live imaging and quantitative approaches—such as FRAP and FCS— challenges remain in understanding how the Bicoid gradient forms, in particular: what processes are determining the Bicoid diffusion? What is the role of the cytoskeleton in modulating Bicoid transport? How do the dynamics depend on the increasing density of nuclei as the embryo develops? How is production of Bicoid temporally regulated?

Quantitative techniques such as FCS and FRAP provide important information about morphogen dynamics, but at different spatial and temporal scales. FCS provides a very local readout of the molecular motion. FRAP, which is typically performed at larger spatial scales than FCS, gives a readout of the effective timescales for cellular-level dynamics, which incorporates multiple processes, such as diffusion, binding, internalization, and degradation. Typically, FCS estimates larger diffusion constants for morphogens than FRAP, as shown with Bicoid in Drosophila [34] and Nodal in zebrafish [37]. A major challenge remains, which is to formulate a robust, biologically-relevant framework that incorporates the different temporal and spatial scales of dynamics into models of gradient formation.

In the *Drosophila* wing disc, Dpp and Wg gradients may also form in an SDD-model-like manner [35]. Yet, alternative transport mechanisms, such as cytoneme-mediated transport via filopodia-like protrusions [38], have been proposed. In this scenario, morphogen transport is more directed than random diffusion. A hurdle still remains, which is to experimentally dissect the modes of morphogen transport in the wing disc, using improved quantitative measurements made by new tools that are necessary to distinguish different mechanisms. From a theoretical perspective, the transport of morphogens by cytonemes has only received limited attention. Further modelling may help to reveal when different transport mechanisms (e.g. random diffusion or directed transport) are favorable. Such modelling needs to account for the cost of generating the cytoneme network, not just the dynamics of morphogen transport through the network.

Models of morphogen gradient formation typically ignore the details of the extracellular space and growth of the tissue. Morphogens do not diffuse freely, but move through a dense microenvironment that restricts their spread [37]. Tissues typically grow in 3D, yet most models of morphogen gradient formation neglect such details. To build up a more precise understanding of gradient formation requires the incorporation of the microenvironment’s structure and dynamic changes in tissue architecture into improved models of gradient formation.

For nearly all morphogens, it is unclear *when* information encoded within the gradient is interpreted and over what time frame such interpretation occurs. Few, if any, morphogens reach a steady state. Therefore, *when* cells read out the concentration is important. How do systems regulate the beginning and end of morphogen readout? Furthermore, how do cells integrate the signal reliably? Temporal and/or spatial averaging are required to reliably interpret the signal [34]. The mechanisms for such averaging are generally poorly understood, particularly in vertebrate systems.

Detailed models have been formulated to describe how downstream genes respond to morphogenetic inputs [39]. There are also many models that describe how morphogen gradients form [18, 34, 35]. Yet, there is currently a paucity of models that incorporate both the *dynamics* of the morphogenetic input and the *dynamics* of the signal interpretation. In particular, one challenge is to develop more realistic models of how downstream genes respond to temporally varying morphogen concentrations.

### 5.3. Advances in science and technology to meet challenges

There have been significant advances in our ability to gain quantitative data on morphogen formation from live embryos. Light-sheet microscopy enables 4D imaging at a sufficient spatial and temporal resolution and with sufficient sensitivity to quantify the formation of morphogen gradients [18]. Light-sheet imaging can also be adapted for FCS, providing spatial maps of morphogen dynamics. Such information can be used to generate more accurate models of morphogen dynamics, which incorporate the microenvironment in which morphogens are transported.

Biological tools, such as endogenous knock-in of fluorescent markers into morphogens, are enabling more accurate measurement of the biologically-relevant morphogen concentration. Tandem reporters, which incorporate two fluorophores with different folding rates, are a powerful tool for measuring morphogen dynamics, as they provide information about the morphogen *age*, and not just concentration, at a particular spatial position [18].

Optogenetic tools allow the exploration of the dynamics of morphogen gradient interpretation. Bicoid combined with the optogenetic molecule Cry2, enables the activity of Bicoid to be switched on or off at specific times and locations [40]. Such temporal control enables the dissection of when and where Bicoid is required for robust interpretation. Similarly, new tools for measuring the live readout of transcription and translation facilitate quantification of the cellular response to morphogen inputs. Such readouts can be used to quantitatively test models of morphogen interpretation.

From a modelling perspective, there is an increasing realization of the importance of incorporating dynamics within models of cell fate specification via morphogens [36]. There have been substantial advances in statistical thermodynamic models and reaction–diffusion models that more realistically incorporate system geometry. With increasing computing power, and higher quality quantitative data for testing and validating model predictions, hopefully theorists will explore the questions of how morphogen gradients form within complex developing systems and how morphogenetic information is temporally interpreted.

### 5.4. Concluding remarks

Morphogens are ubiquitous throughout much of development. The precise formation and readout of concentration gradients is essential. These processes must be tightly controlled in both *space* and *time*. Recent technological advances are giving access to the dynamics in unprecedented detail. With such information, more accurate models of gradient formation and readout can be formulated, which are essential in understanding how tissues reliably and reproducibly generate complex forms.

## 6. Coupling morphogenesis and patterning during amniote gastrulation

### 6.1. Status

**Figure 4.**
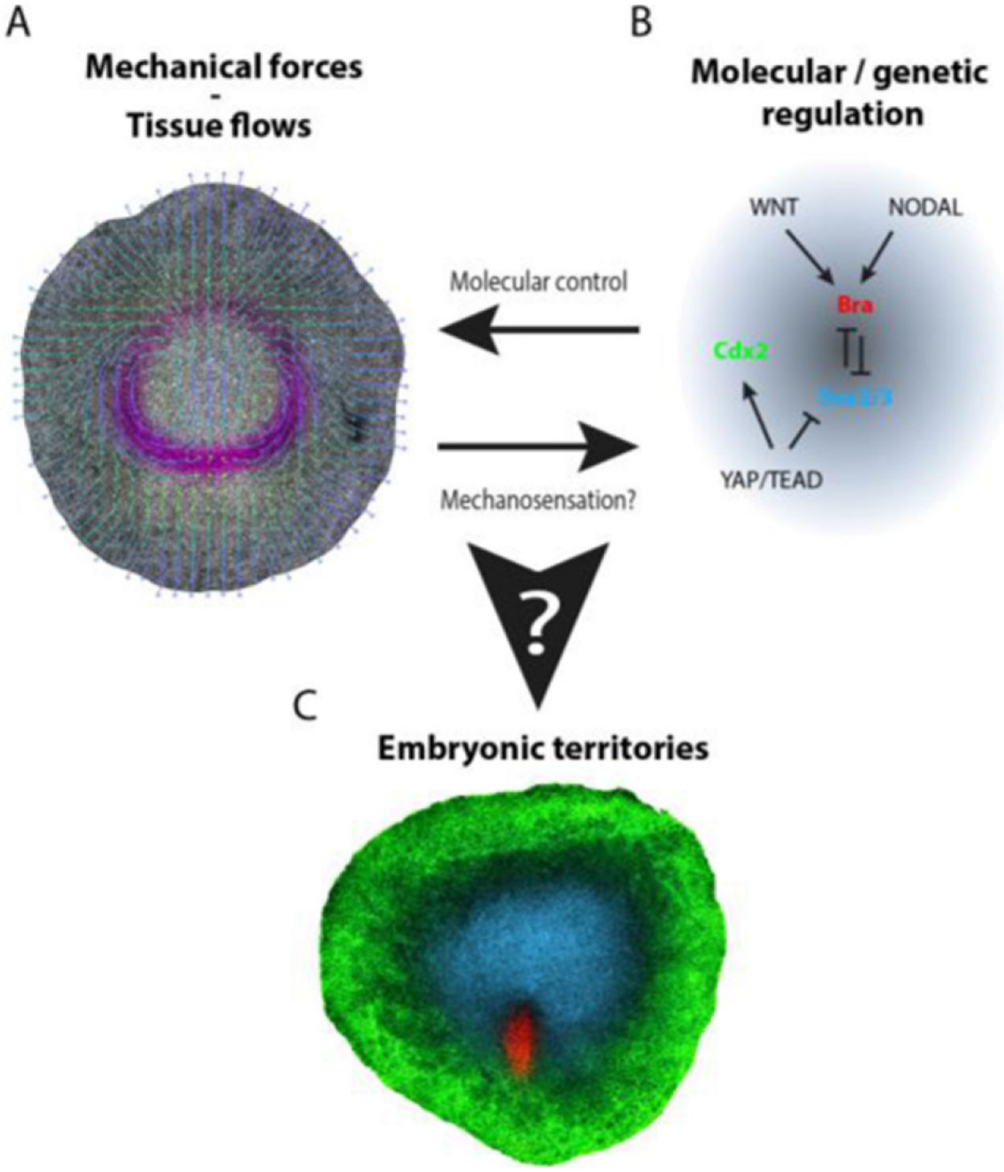
Possible interplay between tissue mechanics (A) and regulatory networks (B) to specify embryonic territories during quail gastrulation (C). Active forces are in magenta in (A); embryonic, mesendodermal, and extraembryonic territories are shown, respectively, in blue, red, and green, in (C).

During vertebrate development, gastrulation is the first major morphogenetic event that lays down the three embryonic germ layers (endoderm, mesoderm, and ectoderm) and the primary axis of the embryo. In amniotes (birds, reptiles and mammals), this process spans several days, during which, the transmission of cell-generated forces remodels the early epithelial embryo into a multilayered organism (figure 4(A)), while signalling and gene regulatory networks concomitantly specify cell fate (figure 4(B)). Whereas the execution of these two tasks must be continuously coupled to ensure the proper regional allocation of cell fate (figure 4(C)), they have mostly been studied independently, arguably because of lack of a suitable experimental model. As a result, little is known about how morphogenetic and patterning programs interplay and coordinate.

Although avian embryos are largely regarded as classical embryological models, the recent resurgence of interest in a physical view of development repositions them as modern mechanobiological systems. Avian embryos are ideally suited for mechanical perturbations and live microscopy, owing to their flat geometry (a two-dimensional epithelial disk), large dimensions (~4 mm diameter) and external development. A striking feature of avian—and more generally amniote—embryos is their highly regulative development, in which cell fates, influenced by inducers and cell-to-cell interactions, can be rerouted by external perturbations to produce the correct spatiotemporal arrangement of patterns. This is remarkably illustrated by the bisection of a gastrulating chicken embryo, which eventually produces two perfectly patterned and shaped embryos [41]. However, to date, the mechanisms underlying such fate redirection remain unclear.

To decipher how mechanical forces and molecular signals might interact to specify or maintain cell fates during gastrulation, it is crucial to obtain a clear view of both its mechanical and molecular control. Decades of genetic, molecular and developmental biology studies have characterized the signalling and gene regulatory networks governing germ-layer specification, highlighting their high level of conservation among vertebrates. For instance, the Wnt and TGF*β*/Nodal signalling pathways have been shown to be critical for mesendoderm specification, whereas the Yap/Tead pathway has been involved in embryonic vs extraembryonic specification. More recently, a clear mechanical picture of avian gastrulation has emerged, showing that the seemingly complex tissue motions that accompany gastrulation can be explained simply by the generation of active forces at the margin between embryonic/extraembryonic territories. The propagation of such forces throughout the embryonic epithelial disk, shown to behave as fluid-like material, then accounts for the observed large-scale, counter-rotational tissue flows [42].

In summary, with only three major cell types, well-defined molecular and genetic regulation, a clear mechanical description, and high amenability to mechanobiological approaches, the gastrulating avian embryo provides a powerful experimental platform to decipher how molecular and mechanical cues combine *in vivo*. Its study is therefore likely to uncover fundamental principles underlying embryonic development in general, with direct relevance to human development.

### 6.2. Current and future challenges

Whereas it has become clear that mechanical forces influence cell fate specification *in vitro* [43], pinpointing an unequivocal role *in vivo* has remained a challenge for several reasons:

- The precise material properties of the gastrulating embryo (e.g. viscosity, elasticity) remain to be characterized and mapped.

This is a prerequisite, not only to obtain an accurate physical description of gastrulation, but also to perform controlled mechanical perturbations. Whereas *in vitro* cell cultures, owing to their great accessibility and simple conformation are well suited for physical measurements using standard apparatus (AFM, cantilevers, pipettes, etc.), similar apparatus must be adapted to accommodate the multilayered organization, culturing conditions and limited accessibility of live embryos [44]. This remains a complex experimental challenge.

- A need remains for robust methods to modulate the forces in living embryos through direct physical intervention, rather than by modulating molecular motors and cytoskeletal components, which may act pleiotropically on signalling pathways.
- Because forces can propagate throughout tissues, much like molecular signals, disentangling the effect of mechanical force vs molecular cues on gene expression is not a trivial task. In particular, changing tissue geometry can affect tissue constraints but also the diffusion of signalling molecules known to be critical during gastrulation.
- Dynamic changes in gene expression, following *bona fide* mechanical perturbations, must be assessed in a quantitative manner to reveal relevant changes.
- Unbiased omics approaches must be designed to identify and characterize mechanotransductive pathways beyond the already well-characterized candidates (e.g. YAP, beta-catenin, etc.) [8, 45].
- To date, a physical description of tissue mechanics has largely been absent from models of gastrulation [46, 47]. As a result, important features such as cell and tissue movements have relied on ad hoc hypotheses and conditions. A real challenge is the elaboration of theoretical models grounded in quantitative descriptions of gene expression and tissue mechanics to obtain testable predictions rather than simple plausibility studies.

### 6.3. Advances in science and technology to meet challenges

The advances required to meet these challenges are diverse, spanning different areas of research. For instance, the field needs input from experimental biophysicists and engineers to design devices that can access the early embryo and subsequently probe and manipulate its mechanical state.

Recent improvements in fluorescent *in situ* hybridization allowing the quantitative and multiplexed detection of single RNA molecules make it possible to measure the expression levels of selected candidate genes (see figure 4(C)). However, whole-transcriptome analysis would open the way to unbiased detection of novel target genes and signatures of previously unidentified mechanosensitive pathways. Single-cell RNAseq analyses have proved successful at characterizing cell fate trajectories for entire embryos, but such approaches disrupt tissue integrity and impose potentially confounding mechanical perturbations. Spatial transcriptomics is emerging as a very promising alternative tool because it offers unbiased sampling, while preserving tissue integrity and enabling the recovery of spatial information [48].

The unambiguous demonstration of the role of forces in controlling gene expression will require the characterization of the underlying mechanotransduction pathways. This will require the development of novel transgenic lines expressing fluorescent reporters and biosensors to read out the dynamics of these pathways. This is necessary to gain actual mechanistic insights and not just information about an ON/OFF state. Surprisingly, despite the well-demonstrated roles of numerous signalling pathways during vertebrate gastrulation and development in general, only a handful of tools exist to report the dynamics of their activities. Using CrispR-Cas9 genome editing to tag endogenous proteins, combined with dynamic approaches and mechanical perturbations, should provide invaluable insights.

Finally, to formulate and constrain quantitative theoretical models capturing the reciprocal feedback loops between tissue mechanics (e.g. regions under compression or tension), dynamics of signalling pathways and cell fate decisions, advances in image acquisition and analysis will be needed. Whereas tremendous progress has been achieved regarding *in toto* imaging and analysis of fly and fish embryos, the application of a similar method to amniotes remains in an early but promising stage [16, 42].

### 6.4. Concluding remarks

Addressing the role of forces in the coupling of morphogenesis and patterning during gastrulation will require skills spanning different areas of physics and biology, from experimental (physical measures) and theoretical physics (conceptual frameworks) to morphogenesis (dynamic tissue-scale analyses), transcriptomics and cellular biology (mechanotransduction characterization) approaches. This will therefore necessitate joint efforts from experts of various backgrounds and students trained in interdisciplinary methods.

## 7. Supra-cellular actin fibre arrays and their role in animal morphogenesis

### 7.1. Status

**Figure 5.**
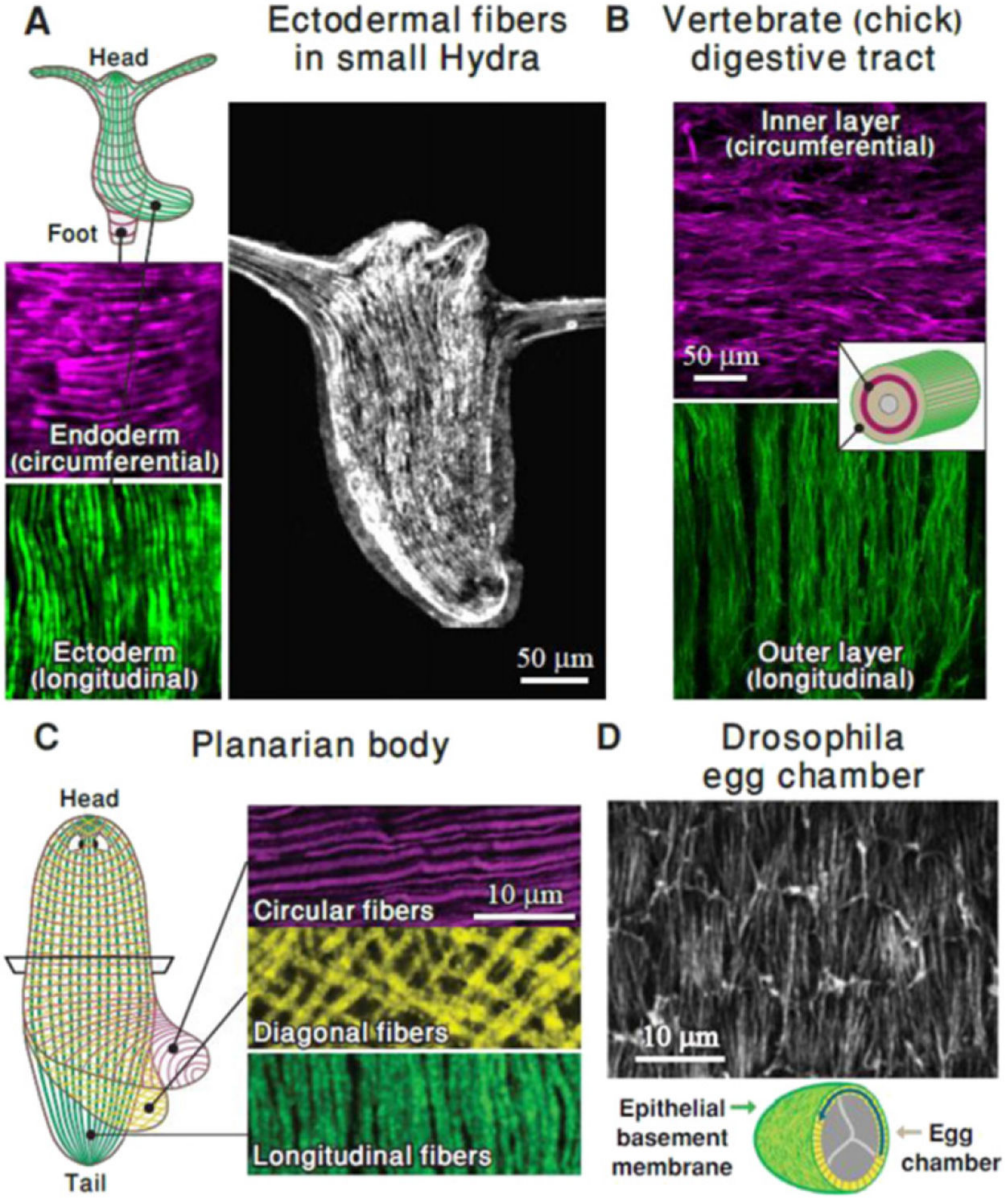
Parallel actin fibre arrays in different model systems. Images and schematics showing actin fibre arrays in (A) *Hydra*, (B) vertebrate gut (courtesy of Tyler Huycke, [53]), (C) planarian (courtesy of Lucila Scimone and Peter Reddien, [50]), and (D) Drosophila (courtesy of Maureen Cetera and Sally Horne-Badovinac, [52]).

Parallel arrays of contractile actin fibres are a common organization theme seen across a variety of organisms, from the simple metazoan *Hydra*, through worms, insects and vertebrates [49-53] (figure 5). Contractile fibres are formed from bundles of actin filaments, bound together by cross-linking proteins, and decorated with myosin motors that generate forces by binding and moving along the filaments. Multiple fibres are arranged into larger-scale structures ranging from cellular arrays of stress fibres, to supra-cellular arrays of fibres formed by mechanically linking actin filaments in neighbouring cells through cell-to-cell junctions. Coordinated motor activity within these structures enables large-scale force-generation and synchronized contraction.

**Figure 6.**
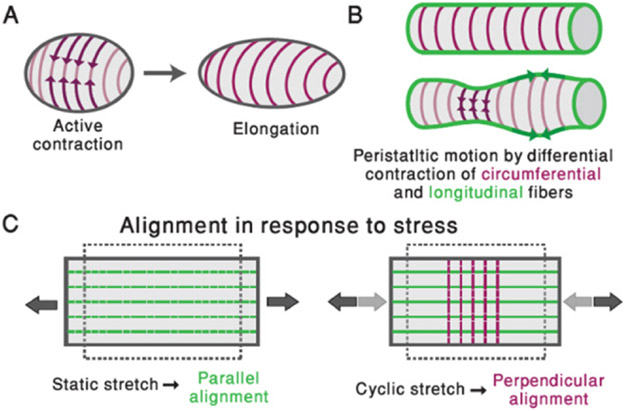
Schematics of actin fibre array function and response to stress.

Actin fibre arrays fulfil important functional roles in patterning during morphogenesis as well as in normal tissue physiology. At a cellular level, aligned stress fibre arrays play a crucial role in controlling cell mechanics and mediating responses to environmental factors such as substrate stiffness, tissue geometry, and external stresses [54]. At a supra-cellular level, actin fibre arrays enable global, coordinated contraction, and provide long-lasting structures that generate and mediate stable, directional mechanical cues. Interestingly, in systems ranging from *Hydra* and planarian to vertebrate smooth muscle and heart, parallel arrays of fibres are often found in two or more adjacent sheets of differing orientation (figure 5), facilitating the production of varying stress patterns which are important for tissue organization and function (figures 6(A) and (B)) [49, 50, 53, 55].

The prevalence of parallel actin fibre arrays points towards some universal principles of organization governing the alignment and stabilization of these structures. *In vitro* experiments on single cells and cell monolayers have shown that fibre assembly and alignment is significantly affected by the mechanical environment. In particular, fibre arrays tend to be oriented parallel to the direction of externally applied static stretching, and at a uniform angle (often perpendicular) in response to cyclic stretching (figure 6(C)) [54]. Theoretically, these responses have been modelled assuming a minimization of elastic energy, incorporating contributions from the fibres as force dipoles, as well as adhesions and/or coupling to a substrate. Interestingly, recent studies of the development of the smooth muscle layers lining the vertebrate gut (figure 5(B)) found a similar response of fibre orientation to internal stress patterns [53]. Once established, these ordered fibre arrays are stably maintained, even in the absence of the stresses required for their initial formation and alignment [53]. In other cases, such as in the alignment of actin fibres at the basal epithelial layer surrounding the Drosophila egg chamber (figure 5(D)), the parallel alignment is reinforced through coordinated motion in which the entire egg chamber rotates along the direction of alignment [52]. While susceptibility to mechanical stress appears to be a common characteristic of actin fibre arrays, little is known about other aspects of their organization. For example, what determines the width of individual fibres or the spacing between them?

The contribution of actin fibre arrays towards patterning and the direction of tissue organization is just beginning to be appreciated. Several examples demonstrate that these arrays can act as scaffolds, both mechanically, by providing structural stiffness in a particular direction, and biochemically, with muscle cells providing biochemical signals that correlate with their global orientation [50]. Furthermore, the contraction of actin fibre arrays can actively drive tissue elongation, or even provide mechanical cues for the formation and alignment of a second, orthogonal array of fibres (figure 6). In *Hydra*, the alignment of actin fibres directs the formation of the body axis in regeneration from excised tissue segments [49, 56], and defects (singularities) in this alignment have recently been shown to act as organization centres for the formation of morphological features [57].

### 7.2. Current and future challenges

Achieving a systematic understanding of the mechanisms underlying the formation of actin fibre arrays during development and their role in patterning morphogenesis is an outstanding challenge. As a major force-generating system, structure and mechanics are intimately coupled in these arrays with substantial mutual feedback [53, 54]. The complexity of this coupling is apparent even at the molecular level, where interactions between cytoskeletal and adhesion components include the formation of catch bonds and other force-dependent processes, which collaboratively, via mechanisms that are still largely unknown, regulate fibre assembly, binding, and turnover. Similarly, at the cellular and supra-cellular level, the assembly and patterning of actin fibre arrays often requires coordinated regulation of cell differentiation, intercellular junctions, and cell–substrate adhesions, but the mechanisms involved are still unclear.

The functional roles of actin fibre arrays in morphogenesis are remarkably diverse, making them difficult to fully discern. Apart from the ability to directly drive changes in tissue shape by coordinated contraction (figure 6), actin fibre organization and contraction can provide mechanical cues, conveyed through either temporal and/or spatial alterations of the stress field, to regulate and coordinate important biological processes across the tissue [51, 53, 57]. Similarly, regular arrays of fibres can provide important positional information for biochemical cues that initiate and stabilize patterns of gene expression [50]. Although both are likely to be essential for morphogenesis, little is known about the mechanisms involved and how the different mechanical and biochemical processes are integrated to lead to robust morphological outcomes.

One of the most profound, yet poorly understood characteristics of actin fibre arrays is the remarkable duality of their regularity and stability on the one hand, and their flexibility and adaptivity on the other [56]. This duality seems essential for their function, providing structural memory and stability to the tissue, but at the same time facilitating their ability to rapidly adapt and drive the significant changes in shape and geometry that are widespread during morphogenesis [49, 56]. How can the same structure be stable over extended timescales yet rapidly remodel when needed? While the mechanisms involved are still unknown, they clearly require complex inter-cellular coordination and feedback, potentially involving additional tissue-scale mechanical processes, such as directed cell migration or modifications of the extra-cellular matrix.

### 7.3. Advances in science and technology to meet challenges

Complementary advances in experimental and analytical tools, as well as the introduction of new conceptual approaches, can greatly enhance our understanding of the assembly, function, and role of actin fibre arrays in patterning morphogenesis. This requires the integration of ideas from physics such as the dynamics of active matter, elasticity, and nematics, with the more prevalent biochemical approaches, as well as making use of the latest computational and mathematical tools.

Techniques for imaging actin dynamics and measuring mechanical properties within developing tissues, without compromising viability or interfering with the developmental process, have advanced considerably over the last decade [58]. Nevertheless, our ability to monitor the mechanical environment *in vivo* and apply mechanical perturbations in a spatially and temporally controlled manner is still limited and hinders our ability to study actin fibre organization and its influence on pattern formation. This is especially important, given the ubiquity of the cytoskeleton in a multitude of cellular processes, making biochemical or genetic alterations of actin fibre arrays extremely disruptive and difficult to isolate, and highlighting the need for tools that enable the controlled, quantifiable and versatile application of forces and constraints to live tissues.

### 7.4. Concluding remarks

As a common organizational structure found across spatial and evolutionary scales, parallel arrays of actin fibres play an important role in directing and maintaining biological structure and patterning. Understanding the universal principles of organization underlying their assembly and function, which involves multiple interacting biochemical and physical processes, will be an important step towards achieving an integrated understanding of morphogenesis.

## 8. Mechano-chemical models for morphogenesis and collective cell migration

### 8.1. Status

Tissue patterning and morphogenesis, as well as the coordination between these processes, in developing organisms rely on the feedback between mechanical and biochemical cues [59]. Similarly, phenomena such as wound healing or tissue regeneration require information to be sensed and transmitted over long lengths and timescales. But the way in which these properties are molecularly encoded and able to emerge robustly across varying scales, in the presence of biological noise, tissue heterogeneity, and extrinsic variability, remains poorly understood.

Biophysical and mathematical modelling has long been used to help bridge this gap. In the early 20th century, D’Arcy Thompson used analogies from material science and soft matter physics to explain the typical hexagonal tiling of epithelia or the shape of spiral horns, thereby providing a mechanical framework to think about morphogenesis. In 1952, Alan Turing proposed a complementary, and highly influential, ‘chemical’ theory of morphogenesis, where he neglected the mechanical properties of tissues to concentrate on how the simple biochemical reaction schemes of diffusing species (termed ‘morphogens’) could give rise to *de novo* patterns, such as the tentacle patterns of *Hydra*. These ideas remained largely theoretical for decades, until advances in genetic manipulation, imaging and mechanical perturbations allowed them to start being tested [60]. However, shape acquisition and cellular patterning are often tightly correlated temporally, rendering an assessment of causation and the underlying mechanism difficult. Indeed, although theories of mechanochemical coupling date back to seminal works by Murray and Oster (among others) in the 80s, or earlier, the way to generically couple mechanical models of tissues with the complex biochemical signalling networks operating in each cell remains an open question [59].

### 8.2. Current and future challenges

Biochemical reaction–diffusion models have proved highly successful in predicting a number of non-trivial features in pattern emergence during development, namely in mouse hair follicles, digit patterning, and early symmetry breaking in zebrafish embryos [60]. These models are highly and elegantly simplified, which is a key strength, as it helps to distill universal principles of patterning, but also raises the key question of whether it is always possible to quantitatively relate the few parameters of the theory (diffusion coefficients, activation/inhibition rates) to biophysical observables [60]. At least three avenues of research are currently being undertaken to help answer these questions.

Firstly, the way in which Turing’s intuition can be extended from two-component systems to the complex biochemical regulatory networks present in cells (typically involving thousands of proteins/genes each) remains unclear, and graph-theoretical methods have recently been proposed as a useful tool to bridge this gap [61], providing insights, for instance, into the importance of diffusible vs non-diffusible species at specific points of the interaction network. This allows for generalizations of classical work seeking to understand complex gene regulatory networks as ‘attractors’, or single-cell states, a perspective made even more topical by the recent advances of single-cell ‘omics’ approaches.

**Figure 7.**
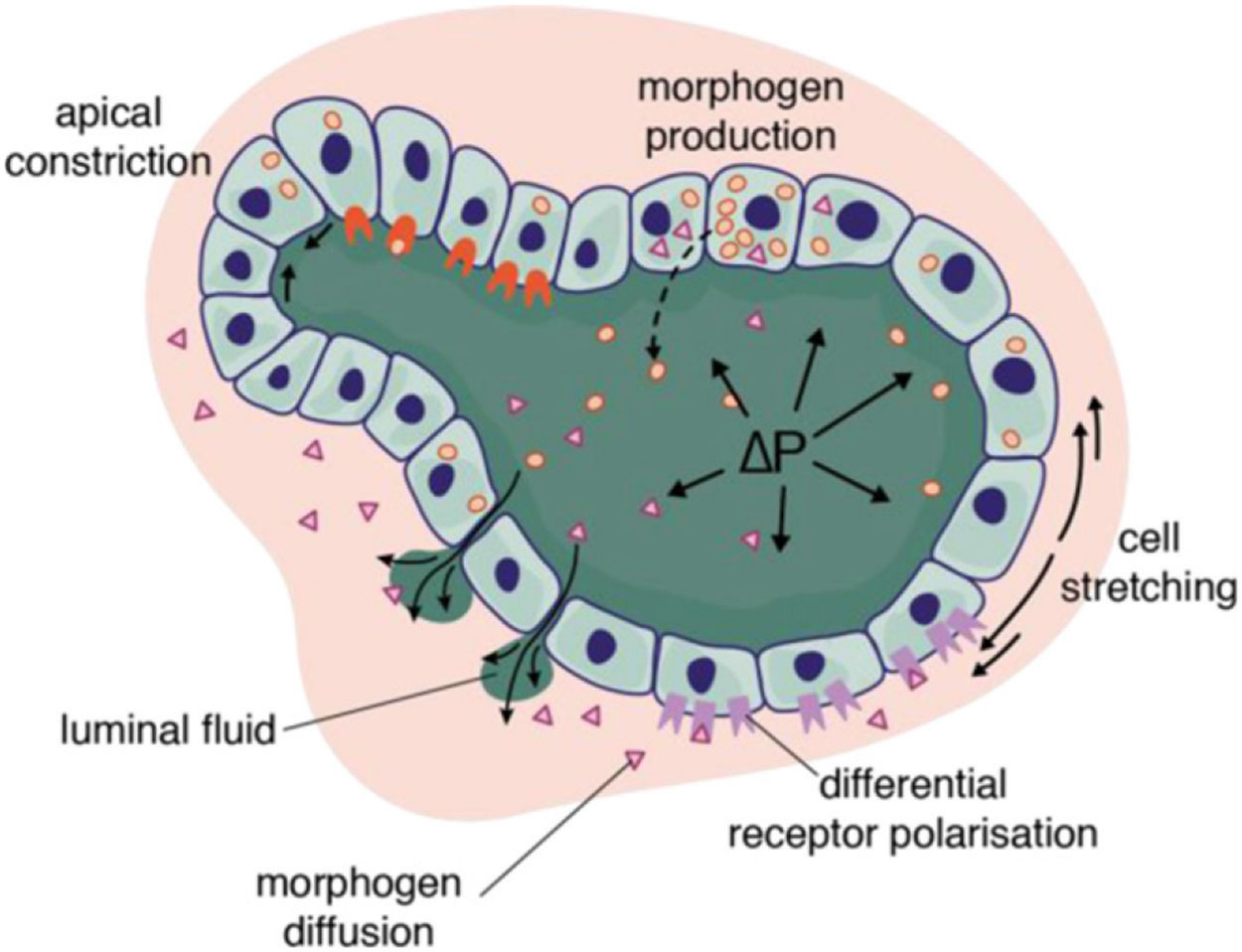
Schematics of possible couplings between geometry, mechanics (luminal pressure Δ*P*, apical constriction, cell stretching, fluid flows etc.) and biochemical signalling (receptor polarization, morphogen production/diffusion) that may drive supracellular patterning and morphogenesis. Art from Claudia Flandoli and modified from [59].

Secondly, exploration of the interplay between morphogen reaction–diffusion and tissue geometry has also started. In cases where epithelial sheets display curvature and signals with a stromal compartment, then a generic coupling exists that tends to modulate morphogenetic fields based on curvature. This has been proposed to occur, for instance, during intestinal morphogenesis [62]. The embryonic intestinal wall consists of an initially flat epithelium of developmental precursors, which evolves in a few days into its characteristic three-dimensional corrugated shape composed of both villi and crypts. In this system, cell-fate specification is required to be tightly coupled to morphogenesis, as stem cells reside in the bottom of crypts, while differentiated cells move towards the top of villi [63]. It has thus been proposed that epithelial cell-derived Shh accumulates in concave stromal regions (i.e. towards the top of villi), due to the geometry of the gut, with the stroma then providing feedback to drive local epithelial differentiation [62]. This allows intestinal cells to break symmetry and adopt their characteristic fate and positional pattern during early development. During subsequent intestinal morphogenesis however, new villi and crypts must be created, which provides a model system to understand how cell fate and tissue shape adapt to growth. Interestingly, this second step involves a surprising degree of cellular plasticity: villi remodelling and fission allow differentiated cells to reposition from villi to nascent crypts, upon which they re-express stem-cell markers, and can make a long-term contribution to the adult organ, as shown via lineage-tracing [63]. In the future, it would be interesting to better understand the mechanisms involved in villi remodelling and the fate plasticity therein, and in particular, whether these epithelial cells are specified by the same principles as those employed during earlier symmetry-breaking [62], as well as understanding what drives their eventual loss of plasticity [63]. Similarly, it has been proposed that the early branching morphogenesis of the mouse kidney arises from a coupling of receptor/ligand dynamics between epithelial and mesenchymal tissues and local-tissue geometry [64]. Such coupling is essential for developmental and patterning robustness [64]. In both intestinal and branching morphogenesis, a next step would be to explicitly include tissue mechanics, rather than implicitly via an imposed geometry, as we discuss in the next paragraph (figure 7).

Thirdly, the explicit interplay between tissue mechanics and reaction–diffusion has started to be explored in the past few years. Understanding this interplay remains highly challenging, due to the number of possible couplings to consider. Interestingly, however, coupling the two has been found to facilitate patterning by lifting restrictive conditions on the network structure required for Turing patterns, but also by producing qualitatively different instabilities. Recent theoretical efforts include combining three-dimensional vertex models of epithelial mechanics with morphogen diffusion, assuming certain mutual feedback loops between cytoskeletal contractility, cell shape and morphogen concentration [65], or incorporating the biphasic, porous nature of three-dimensional tissues, assuming feedback between morphogen concentration, fluid flows and cellular growth [66]. Strikingly, a few experimental examples of *bona fide* mechanochemical patterning have started to emerge in the past few years. This includes collective epithelial cell migration towards a wound *in vivo* or a free edge *in vitro*, where complex oscillatory patterns of stress, traction forces and cell density have been observed, and have been shown to be accompanied by similar patterns of biochemical signalling, such as ERK/MAPK activity or YAP/TAZ nuclear localization, two key mechano-sensitive pathways. Similarly to a mechanochemical loop, the disruption of ERK/MAPK patterns by the over-activation or inhibition of ERK also disrupted density patterns, long-ranged cell polarization, and efficient wound healing. ERK/MAPK signalling, traction forces, and cell density thus feed back to each other, giving rise to mechano-chemical waves which allow epithelial cells to break symmetry and display long-range order in their collective migration towards wound edges [67]. This raises the possibility that mechanochemical waves could help to transmit information across long distances, an intriguing concept to explore in other settings.

### 8.3. Advances in science and technology to meet challenges

Besides further theoretical research in systems biology, biophysics, and active matter, further advances will require the combination and development of a number of experimental tools. Firstly, spatiotemporally accurate measurements of mechanical forces *in vivo* remain a challenge. This has, nonetheless, started to be alleviated by improvements in two-dimensional and three-dimensional force-inference methods (allowing force fields to be inferred from high-resolution microscopy images), as well as by direct-measurement tools, such as micropipettes for cellular tension, micro-pressure probes for hydraulic pressure, or droplets for mapping local stresses in three-dimensional tissues. Secondly, improved live measurements of intracellular protein activity (such as fluorescence resonance energy transfer (FRET) or kinase translocation reporters (KTR) systems), and reporters of cellular fate with high temporal precision will be needed to correlate biochemical activity with the local geometrical/mechanical state. Thirdly, progress in spatial transcriptomics and inferring temporal information from single-cell sequencing datasets will also be critical to achieve a more global ‘systems view’ of these processes. Precise perturbation tools, such as optogenetics and microfluidics will also be needed to progress from correlations to causation. Lastly, advances in organoid/explant research (see contribution from Heemskerk *et al*) will also offer an exciting new platform to address many of these questions in more experimentally tractable settings that are compatible with high-resolution live imaging. It is striking, for instance, that although some systems such as intestinal organoids display a similar correlation between fate and shape as *in vivo*, other models, such as gastruloids, show precise fate specification despite divergent morphogenetic events [68].

### 8.4. Concluding remarks

Understanding this will require truly multidisciplinary collaborations involving biologists, engineers, and physicists, to develop new theoretical frameworks for mechano-chemical self-organization, as well as testing these frameworks via combinations of live imaging, optogenetics, force manipulation, organoid culture, and microfluidics.

## 9. Guiding the formation of tissue structure through self-organization

### 9.1. Status

Tissue engineers strive to build tissues with complex structures from simple cellular building blocks. This goal requires an understanding of how tissue self-organization emerges from the molecular and physical properties of different cell types. An important step towards this goal was Yoshiki Sasai’s first report of stem-cell-derived organoids, including an optic cup and a layered neural cortex, that self-organized *in vitro* [69]. Through an analysis of the molecular and physical processes contributing to organoid formation, Sasai articulated several general principles for guiding the formation of tissues through self-organization [70]. Among them are: working with the molecules and forces that drive tissue formation during development (rather than imposing constraints on the cells from the top down); directing tissue formation beginning with cells in the correct molecular and physical state; and arranging cells in a manner conducive to their self-organization by using the appropriate cell numbers, cell-type composition, relative cell positions, and boundary conditions. Sasai noted that deviating from these principles would lead to undesirable or heterogeneous outcomes. His efforts anticipated many of the challenges tissue engineers currently face as they work to more efficiently guide the formation of tissue structure through self-organization.

### 9.2. Current and future challenges

**Figure 8.**
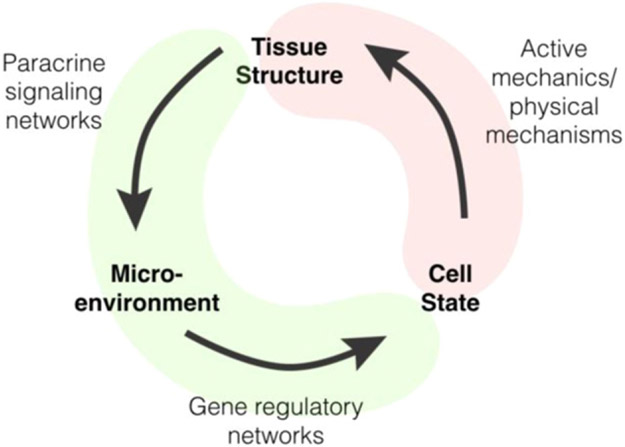
Tissue structure, defined as the composition, shape and three-dimensional arrangement of cells and extra-cellular matrices, arises through programs of self-organization. Tissue structure is linked to cell state through its impact on the microenvironment (green). The microenvironment is the sum of each paracrine signal impinging on a given cell, weighted by constraints provided by the local tissue structure. Active cell mechanics and other physical processes emerge from the molecular state of a cell and its ability to dissipate energy (red). Cell-generated forces result in shape changes and cell rearrangements that underlie many aspects of tissue-structure formation.

Tissues assemble from living cellular building blocks whose physical properties can be altered by self-organization itself—a phenomenon unfamiliar to scientists and engineers who work with non-living building blocks such as atoms and molecules. Imagine the surprise of an organic chemist upon finding that carbon spontaneously turned into nitrogen in a specific chemical microenvironment. Unlike atoms, the physical properties of cells are plastic, and cell identity can change in different microenvironments. In adult tissues, structure, microenvironment, and cell identity mutually reinforce each other, resulting in a robust and steady-state tissue structure (figure 8). In developing tissues, however, structure, microenvironment and cell identity do not reinforce one another, resulting in continuing changes to tissue structure. In the context of development, changes in the cell state lead to corresponding changes to cell mechanical properties. Forces become out of balance resulting in changes to tissue structure, altering the lines of communication between cells and modifying each cell’s microenvironment. New microenvironmental signals are interpreted by gene regulatory networks to determine whether to further change the cell state. This cycle of structural changes, microenvironmental changes, and cell-state changes continues until the tissue arrives at the adult steady state.

Since cell state is closely linked to the outcome of self-organization, a challenge for guiding tissue formation is initiating the process with cells of the correct physical and molecular properties. Isolating populations of single cells from developing tissues at the proper stage is one approach to this challenge, but it is problematic because the appropriate cellular characteristics may only transiently exist outside the correct microenvironment. An alternative is to derive these cells *in vitro* by exposing stem or progenitor cells to sequences of molecular, physical, and electrical cues. However, this approach is hampered because the relevant cues are often unknown. Without better mapping between the microenvironment and the cell state, it will be difficult to systematically isolate cells capable of robust self-organization.

The incomplete understanding of how the molecular state maps to the mechanical state poses an additional challenge. The same mechanical property, such as the quantitative value of cell cortex tension, can arise in different cell types within different gene-expression programs. Thus, even if it were possible to specify the molecular state of a cell by engineering its microenvironment or genome, the particular state to programme to dial in a desired physical property would still be unknown.

Controlling the initial positions of cells in 3D represents yet another challenge. Cells actively respond to their immediate surroundings when determining their next move. Thus, self-organization can progress along different paths depending on the cues that cells receive from their immediate surroundings, a phenomenon referred to as stigmergy [71]. Therefore, the initial composition and arrangement of cells, extracellular matrix molecules, and other components of a tissue can have a major influence on the outcome of self-organization, leading the same cells to occasionally organize into different three-dimensional structures when initially arranged in different positions.

Similarly, the boundary between a population of self-organizing cells and the surrounding environment profoundly affects the outcome of self-organization. In particular, spurious mechanical cues delivered to self-organizing tissues by their boundaries can lead to undesired outcomes, such as the scattering of cells towards a stiff interface or the inversion of tissue structure due to a missing mechanical interface [72]. *In vivo* interfaces change their shapes and physical properties along with the tissues themselves. Control over the dynamics of interfaces *in vitro* has largely eluded tissue engineers and represents yet another challenge.

Beyond these technical challenges, tissue engineers lack generalizable principles to predict how living, non-equilibrium structures change in time to arrive at a steady state. Chemists and materials scientists rely on quantitative theories and laws, including statistical mechanics and kinetics, to organize their observations and predict how non-living matter self-assembles. However, it is not clear whether these theories break down when abstracted to non-equilibrium, or ‘active,’ materials at the cell-to-tissue scale [73]. The way in which these models must be modified to account for cell-to-cell interactions and state changes adds yet another wrinkle [74]. Without foundational principles, our ability to guide self-organization will be slow to progress.

### 9.3. Advances in science and technology to meet challenges

Numerous opportunities exist for scientists and engineers to further our understanding and control of self-organization. In order to programme cells efficiently, methods are needed to identify the physical and molecular cues in the microenvironment that direct cell state. *In situ* methods, such as spatially resolved transcriptomics (e.g. Seq-FISH [75]) and proteomics (e.g. multiplexed ion-beam imaging [76]) represent the state of the art. However, these methods may be better at inferring cell state as a function of cellular neighbourhood than at identifying the specific molecules or forces responsible for directing cell state. Efforts to recreate specific microenvironments and then measure their impact on transcription or translation may offer a complementary approach.

Along similar lines, progress in our ability to perform simultaneous molecular and physical measurements on single cells will advance our ability to link the molecular state of a cell with its active material properties. Achieving these measurements will likely require new devices for performing these distinct modalities of measurement. Additional work is necessary to make these measurements *in situ*, and to infer the physical properties and forces generated by groups of cells within living tissues.

Development is a multistep process, and at every point the structure of tissues and their boundaries are arranged so as to set the stage for the next step. Therefore, methods are needed to specify the initial structure of a tissue and its boundaries in 3D culture so as to prepare for its subsequent self-organization. Printing and other top-down technologies have been the focus for specifying initial conditions, but they typically lack the spatial resolution to arrange single cells appropriately and do not scale when many copies of a tissue are required. Synthetic biology methods may provide an alternate path forward. For example, orthogonal genetic programs that direct self-organization could be used to programme the initial conditions of cells in culture, after which the synthetic programs would be terminated, thereby allowing the cells to continue self-organizing autonomously [78].

Dynamic tissue boundaries are fundamental to living materials, but do not have obvious parallels in the non-living world. Therefore, recreating boundaries with the necessary properties *in vitro* poses a major challenge. Materials scientists are working to build boundaries from dynamic materials that undergo modest changes to shape or stiffness when exposed to different stimuli, but these materials cannot mimic the feedback between a tissue and its boundary that occurs in living organisms. The best solution to this challenge will begin with studying the properties of living boundaries *in vivo*. Areas for immediate focus include the way in which the interface between parenchyma and mesenchyme is assembled and maintained, as well as how adventitia and serosa support the back side of the mesenchyme [77]. Understanding the properties of these tissue interfaces, and recreating them *in vitro*, will advance our capacity to guide tissue formation more efficiently and across larger length scales.

Finally, the theoretical underpinnings of tissue self-organization are ripe for exploration. In particular, basic research into the properties of active matter at the cell and tissue scale is called for. Coupling these studies with dynamic systems theory will be necessary to incorporate information exchange and cell state transitions. Whether unifying principles exist will only become clear as these efforts mature.

### 9.4. Concluding remarks

An improved ability to guide tissue self-organization will unleash new applications and spawn new industries. However, realizing this potential will require important advances in science, technology, and theory. These challenges represent exciting opportunities for young scientists and will require an interdisciplinary effort.

## 10. Phase transitions, spatial control of mRNA localization and gene expression in syncytial fungi

### 10.1. Status

Compartmentalizing cytoplasm in both time and space is critical for cell function. In the last ten years, membraneless organelles, also known as biological condensates and droplets, have been shown to compartmentalize the cytoplasm. These compartments exhibit liquid-like properties and are thought to form through a process known as liquid–liquid phase separation (LLPS), in which a mixed solution separates into concentrated droplets and a dilute bulk phase. LLPS is driven by weak, multivalent interactions between proteins, often with disordered domains, and nucleic acids, such as RNA. Because these condensates are enriched for certain proteins and nucleic acids, they are predicted to increase reaction rates, sequester molecules, and serve as signalling or scaffolding hubs [81]. However, there are still limited examples of specific biological functions that require condensation. In syncytial fungal cells, which contain hundreds of nuclei and multiple sites of polar growth within a single cytoplasm, compartmentalizing the cytoplasm is particularly important. Biological condensates perform a role in localizing and regulating RNA to spatially and temporally organize syncytial cells. In fungi, RNA containing condensates are important for polar growth, symmetry breaking, nuclear cycling, and mating-type switching [82].

A number of unresolved questions remain about how biological condensates function in cells, but thanks to the incredibly rapid advance of this field in the last decade, some of these problems can now be addressed.

### 10.2. Current and future challenges

One challenge for the field is to determine how ubiquitous phase separation is in cells. Whether phase separation is a common phenomenon across the proteome, as seems likely, or is limited to a small number of proteins, will likely be answered in the next decade. Another major challenge is to understand the mechanisms by which condensates actually influence specific biochemistry [84]. There is evidence of changes in reaction rates within condensates but the examples for molecular function are still quite limited [84]. For proteins and RNAs found to form biological condensates, it will be important to answer several additional questions.

#### How are condensates regulated in time and space?

Biological condensates must form at, or be transported to, a specific location in the cell, and dissolve when necessary. The role of regulators that can deliver specific post-translational modifications that may alter valency and interaction strengths among key scaffolds in a condensate are likely critical and most await discovery. In addition, that way in which cells regulate the material properties of condensates including size, surface tension, viscosity, elasticity, pore size, etc., is not understood. The study of free-living cells that cannot regulate their internal temperature or control their environment will be especially useful for understanding how the physical chemistry and biochemical control that underlies the formation of condensates are sensitive to, or exploit, these physical parameters to cope with extremes in the environment.

#### How is specificity achieved and maintained?

The formation of biological condensates is predicted to occur through a generalizable thermodynamically-driven process involving many low-affinity interactions. Many phase-separating proteins share features such as low sequence complexity and a predicted intrinsically disordered structure. How are specific combinations of proteins and RNAs brought together while excluding others, despite seemingly similar features? There are still very limited models for how protein affinity may be encoded at the scale of individual amino-acid composition in low-complexity sequences. Understanding how different droplets establish and maintain their individual identities, rather than intermixing, is a crucial question in the field.

#### How do biological condensates affect RNA dynamics in cells?

Many biological condensates are known to contain RNAs but, with few exceptions, their functions are not well understood. Learning whether specific condensates promote or prevent translation, degradation and transport, and under what conditions, will help us understand both basic cell physiology and dysregulation in disease.

### 10.3. Advances in science and technology to meet challenges

Only in the last ten years has the concept of LLPS, a well-described phenomenon in chemistry and physics, been applied to biological systems as a way to understand the spatial patterning of the cytosol. Since then, the number of proteins believed to undergo some form of phase separation, either *in vivo* or *in vitro*, has grown from zero to hundreds. As the number of proteins that undergo LLPS has increased, a variety of computational models, from particle-based molecular-dynamics simulations to continuum models, have been developed to predict the properties of phase-separated systems and the behaviours of individual molecules within the system [79]. A major challenge in all of the current modelling approaches is to capture the complexity of molecular composition and interactions in an experimentally accurate and computationally feasible manner.

To ground these models and better understand how condensates function in cells, it will be necessary to measure the physical properties of the droplets and of the cellular milieu in which they form. Unlike traditional membrane-bound organelles, droplets are dependent on the physical environment around them and are sensitive to changes in pH, temperature and physical crowding [88]. The recent creation of *in vivo* probes to measure the mesoscale viscosity of cytoplasm, complemented by new approaches for manipulating elasticity and compression *in vitro*, will be critical new tools [80]. In addition, advances in microscopy, such as FCS, allow the measurement of concentration and diffusion rates within droplets to a high degree of accuracy [79].

One key question is whether the physical properties of droplets, for example their viscosity, are important for their function. One way to experimentally alter the properties of condensates is by changing the temperature of the system. Because fungi are able to tolerate temperature changes, they are an excellent model to test these questions. By using these new tools to carefully measure the properties of condensates and measuring the functional consequences, it will most likely be possible to link the material properties of condensates to their functions in cells.

Recent research combined with new tools has shed light on how identity is maintained in some condensates. One critical factor in droplets that contain RNAs is the RNA itself. In the case of Whi3 droplets in *Ashbya gossypii*, distinct condensates with functions in the cell cycle and cell polarity are composed of the same protein but contain different RNA [83]. In this system, the functionally distinct droplets seem to arise from specific RNA-to-RNA interactions that join or segregate different RNAs, which may then recruit distinct protein components in addition to Whi3. This depends, in part, on the sequence of the RNA, but also on the RNA structure, which appears to scaffold the two droplet types such that they do not mix [83]. New tools developed to probe the structure of long RNAs through structure-dependent mutagenesis will allow researchers to test the universality of this phenomenon [86]. In addition to the role of RNA in conferring identity, structural modelling and mutagenesis has suggested ways in which ‘hidden order’ within unstructured domains in proteins may convey specificity to these droplets [79]. These advances in studying the structures of RNA and protein within droplets will allow researchers to probe how droplets maintain their identities, and to better understand ways that identity can be lost in disease states.

The application and evolution of tools to visualize RNA also now make it easier to define the functions of condensates in mRNA regulation. Researchers can now monitor RNA in living cells and in real time. For example, stem-loop-binding proteins, such as MS2, have been used to fluorescently label single mRNAs *in vivo* and to track their transcription, movement, and degradation within the cell, or to monitor protein translation from an mRNA [87]. Using these techniques, it is now possible to assess how condensates affect RNA localization, stability and translation. Additionally, optogenetics now allows researchers to generate and dissolve condensates experimentally, allowing new ways to test for functional relevance [85].

### 10.4. Concluding remarks

The field of biological phase separation has exploded in the last decade and may provide a unifying explanation for many phenomena in biology. Large multinucleate cells such as syncytial fungi provide opportunities to discover new and surprising mechanisms of cytoplasmic organization, and may ultimately provide insights applicable to other cell types such as neurons. As the field continues to mature, it will be critical to move from phenomenology to mechanistic descriptions of condensates accompanied by *in vivo* measurements and phenotypes. New technologies and modelling approaches make these goals feasible and ensure an exciting future for the field of biological phase separation.

## 11. The role of mechanics in a ‘post-molecular’ conception of morphogenesis

### 11.1. Status

In recent decades, the study of morphogenesis has been dominated by molecular interrogation. Nobel prize-winning work on *Drosophila* conducted in the early 1980s used mutagenesis screens to identify individual genes that were essential for proper morphogenesis [89]. To the surprise of many who believed that higher organisms built their complex morphologies with genes not present in simpler organisms, the genes discovered in flies were found to be conserved across animals. The development of mouse genetics in the 1980s and thereafter allowed for an investigation into the essential roles of these genes in vertebrate development. More recently, the exponential improvement of sequencing technologies has allowed for an expansion of molecular characterization, culminating in current-day single-cell sequencing.

This molecular focus has highlighted the importance of two classes of genes that have been viewed through distinct conceptual frameworks. The first are transcription factors, which are sufficient to bring about widescale changes in gene-expression states as tissue morphology increases in complexity. Remarkably, the forced expression of small numbers of transcription factors can reprogram adult cells to an embryonic stem cell state [90]. Transcription factors are central to gene regulatory networks, which have been the main framework for explaining the successive emergence of cell fates [91]. The second class of genes performs coding for signalling molecules and their pathway components, which allows cells to communicate with each other. The dominant frameworks, most notably reaction–diffusion systems and morphogen gradients, posit that the diffusion of such signalling molecules across tissues creates molecular patterns that serve as a blueprint for morphology [32, 92].

Despite a saturation in the identification of the molecular components critical for morphogenesis, a mechanistic explanation of how tissues are physically constructed still remains beyond our grasp. Such a gap is made apparent in organoid cultures, where appropriate fates can be reproduced but control over morphology remains primitive. Studies focussed on mechanics are now appreciated as essential to addressing this gap. A fundamental challenge is to integrate mechanical perspectives with existing molecular frameworks that have shaped inquiry for decades. Here, we propose a path forward to achieve this goal and recommend that the integration of mechanics will require some revision of the molecular conceptions of morphogenesis.

### 11.2. Current and future challenges

While mechanical processes are crucial to morphogenesis, it is currently unclear whether key mechanical processes will be conserved across model systems, as in the case of master genetic regulators. For instance, do the mechanics of *Drosophila* embryos map to those necessary to generate human tissues, the latter of which occurs at a scale that is orders of magnitude larger than the former? Given this uncertainty, it may be critical to select contexts in which the study of mechanical processes have a strong likelihood of uncovering principles generalizable up to humans. In vertebrates, the most morphologically conserved stage of development is the onset of organogenesis, known as the phylotypic stage. Here, the remarkable similarity in embryonic shape and size suggests the presence of mechanical processes that are so constrained that evolution cannot develop alternate ways to generate such morphologies. Any elucidation of phylotypic-stage mechanics has the power to unlock a deeply foundational aspect of morphogenesis.

**Figure 9.**
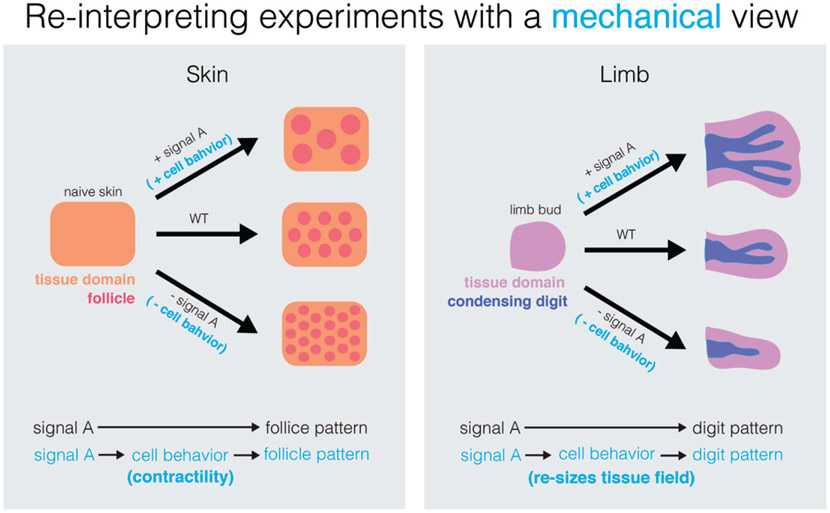
Experiments where tuning the levels of signalling molecules shifts patterns have been interpreted as evidence that the spatial gradient of signals sets up a molecular blueprint. What has not been taken into account is that such signals most likely modulate the physical behaviours of cells, which in turn changes the manner in which cells come together to construct the pattern.

The molecular characterization of the onset of vertebrate organogenesis has revealed a striking array of signalling molecule and transcription-factor expression patterns across developing tissues. Molecular patterning models have typically assumed that these patterns determine the arrangements of emergent morphological features ranging from the digits of the limb to the branches of the lung and the follicles in the skin [93-95]. For example, the vertebrate limb is a system in which morphogen gradient models have framed an understanding of digit-pattern formation. Removing or blocking Sonic hedgehog (Shh) results in a loss of digit number, whereas an ectopic source of Shh opposite to the endogenous source causes mirror-image digit duplication. These results, seen through morphogen models, have been taken as evidence that a spatial gradient of Shh concentration sets up a molecular blueprint for digits. However, a broader perspective that takes mechanics into account during these perturbations reveals that the entire limb field changes size, depending on Shh exposure. Ablation of Shh shrinks the size of the limb paddle (hand region) whereas ectopic sources double its size. Thus, the impact of Shh may be to modulate the physical size of the limb, which in turn changes the resulting limb pattern (figure 9).

**Figure 10.**
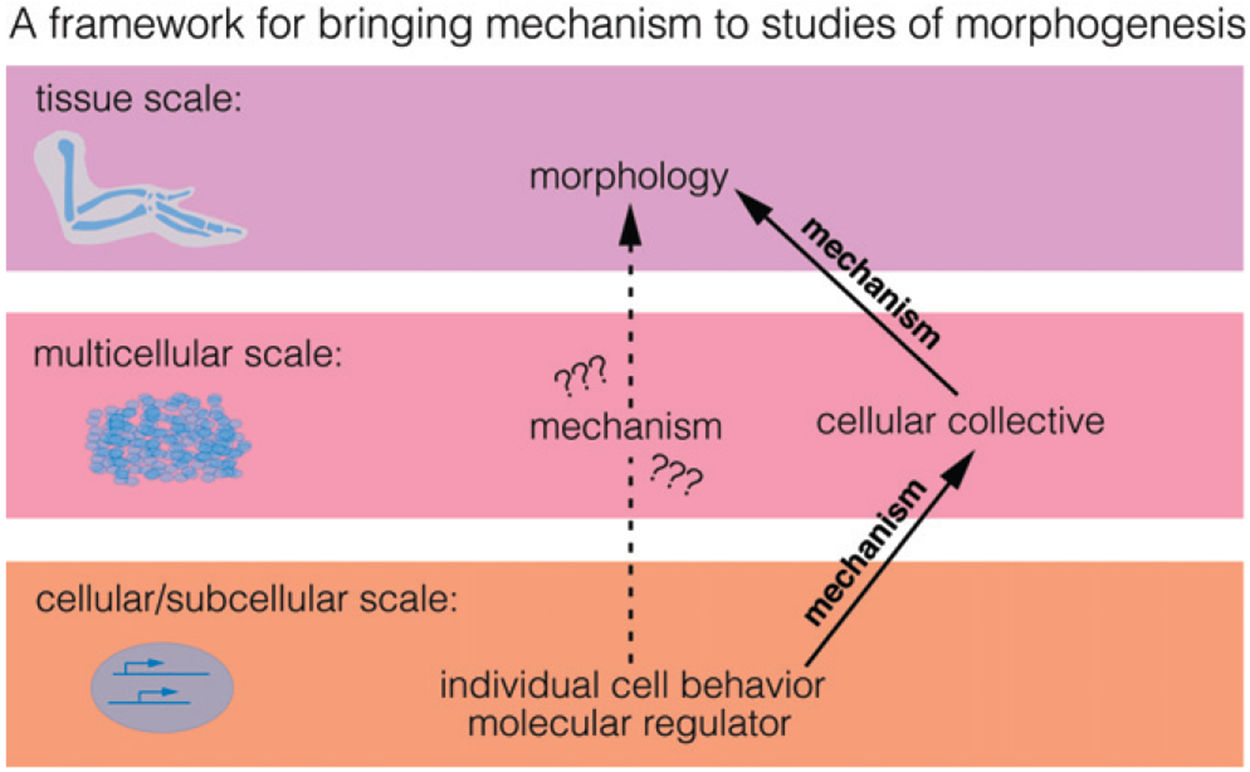
Morphogenesis involves critical events across scales. Mechanical aspects of multicellular mechanics may serve as a bridge between molecular regulators and tissue-level phenotypes.

Much like the limb, studies of the developing skin have focussed on the patterning role played by diffusing signalling molecules. In the mouse, the experimental characterization of Wnt and DKK signals was interpreted via reaction–diffusion models [95]. Based on these models, it was assumed that periodic spatial patterns, after being generated, conferred information to cells to generate properly sized and spaced follicles. Thus, the addition of ectopic signals to skin cultures was assumed to have the primary effect of tuning the molecular pattern landscape. An alternate interpretation, when mechanics is taken into account, is that changing signals directly alters the physical behaviour of cells, which in turn shifts the collective dynamics to change the follicle pattern (figure 9). Moving forward, a central challenge in the incorporation of a mechanical view is that it necessitates a reconsideration of the conclusions of prior molecular studies, many of which may have neglected to account for the mechanical changes that may result from molecular perturbations (figure 10).

Given that physical events occur at every length scale from the atomic to the tissue, another challenge is to select the length scales at which mechanics shape emergent morphological patterns. While the mechanics of individual cells may be the most tractable scale, due to the wealth of insight and technology from the field of cell biology, it is unclear whether such insights can be extrapolated to understand the mechanics of collectives of cells inherent in tissue formation. Instead, new tools and concepts must be developed to understand mechanics at the scale between single cells and a whole tissue—that of the multicellular collective.

### 11.3. Advances in science and technology to meet challenges

While more effective measurement and perturbation of physical interactions across collectives of cells will be essential, the fundamental bottleneck is the need for a conceptual framework that can account for the molecular observations made over the past several decades but also incorporate the role of mechanics. Such a conceptual approach is illustrated in a recent study on the initiation of follicles in avian skin [96]. This work suggests a process of morphogenesis that is dependent on the multicellular self-organization of skin cells into dense clusters in order to initiate follicle morphology. The formation of these clusters also directly activates a mechanotransductive pathway that initiates the gene expression state of follicles.

A number of potentially generalizable features can be taken away from this mechanism. First, symmetry can be broken in the absence of a molecular pattern through mechanical events that occur in an *emergent* manner at the multicellular scale [97]. Second, due to their emergent character, these processes play a *regulatory* role in determining morphology and are not merely an elaboration of molecular instructions. These features suggest that molecular pre-patterns need not be necessary to initiate tissue morphologies. Instead, (many of) the molecular patterns that have been observed in tissues for decades may be the direct consequence of mechanical impulses communicated from the multicellular scale to the genomic level. Such communication could be one of the critical roles played by the increasingly appreciated mechanotransductive pathways. Therefore, patterns of gene expression must be considered as modulators of cell mechanics that then feed upwards to tune multicellular mechanical processes that are already in play through their own emergent accord.

### 11.4. Concluding remarks

If vertebrate organogenesis is governed by mechanical processes at the multicellular scale that are not reducible to molecular level regulation, cellular- and molecular-level analysis alone will be insufficient to fully grasp morphogenesis. Instead, we anticipate that a ‘post-molecular’ conception of morphogenesis that focuses on multicellular mechanics will be the key to link the decades-long analysis of molecular regulators to tissue-level morphology. As has been shown in the case of theoretical studies of cellular automata, where very simple local rules, when iteratively deployed, are capable of generating tremendous richness of pattern, multicellular mechanical processes may be driven by simple cellular behaviours that nonetheless generate complex final forms. Despite the importance of mechanics for the future of studies in morphogenesis, it is critical to maintain sight of molecules and regulation of the genome. In this new conceptual regime, however, molecules, rather than ‘encoding’ for morphological patterns in any direct sense, are likely to allow for a more robust deployment of the emergent mechanical forces that sculpt tissues at the multicellular scale.

## 12. Dynamic interplay between cell shape and division positioning in the morphogenesis of early embryos

### 12.1. Status

Cell division is pivotal to tissue morphogenesis, cell-size control and fate specification. Even when embedded in complex tissues, cells divide with a predictable position and orientation. This has long fascinated life scientists, with the formulation of empirical rules for division site specification dating from as early as the end of the 19th century (reviewed in [98]). The choreography of reductive blastomere divisions that mark the development of early embryos, known as cleavage patterns, is a striking example of regular cell-division geometries. Because blastomeres do not migrate, grow or die, the morphogenesis of the embryo and the position of each cell will solely depend on the history of their divisions and adhesion forces between cells [99]. Cleavage patterns come with remarkable diversity, from orthogonal, holoblastic, meroblastic to spiral patterns. To date, however, their regulation remains poorly understood. This is in part because eggs are extremely large, often opaque, and thus difficult to image; and also because genetic manipulations are limited by the large pool of maternal proteins and mRNA. Yet, studying cleavage patterns is of profound interest for the field of cell division and morphogenesis. First, it provides a dramatic physical limit to cell division, with very large cells that must divide rapidly and accurately. Second, the diversity of cleavages offers a rich repertoire of phenotypes to study division positioning and tissue morphogenesis in 3D.

### 12.2. Current and future challenges

#### 12.2.1. Mechanisms of microtubule force generation for division specification

**Figure 11.**
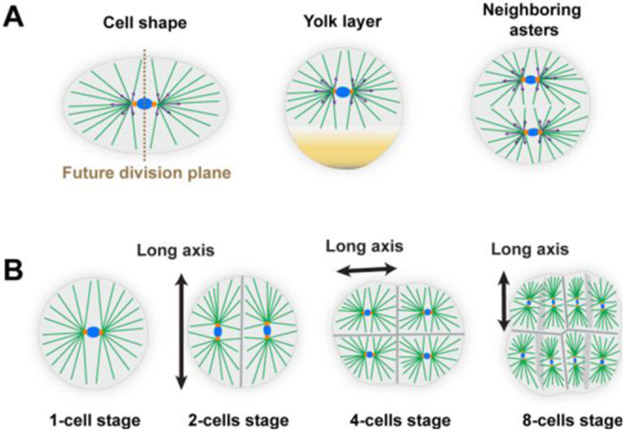
(A) Factors that affect the shape of MT asters in early embryos, and thus contribute to position and orient centrosomes and cell divisions through length-dependent MT forces. (B) An iterative cycle of cell shape influencing division position and division influencing subsequent cell shapes and thus division orientation in the next cycle.

The regulation of division positioning has been studied in many model systems, including yeast and adherent vertebrate cells. In most cells, the nucleus and/or mitotic spindles are first positioned and oriented by forces and torques emanating from astral MTs, and cytokinesis then bisects the spindle axis in anaphase. In small somatic cells, astral MTs are generally thought to be pulled upon by dynein motors attached to the cell cortex [98]. In contrast, the dominant model in large eggs and early blastomeres is that division may be primarily organized from forces exerted upon MTs directly by bulk cytoplasm. This is best demonstrated by recent work recapitulating the division patterns of frog embryos from bulk cytoplasm extracts, which lack cortex and plasma membrane [100]. This essay highlights the current view of the regulation of symmetric and oriented divisions in many early embryos, including those of echinoderms, ascidians, fishes or frogs [99, 101-103]. In this view, dynein pulling from bulk allows longer MTs to generate more pulling forces, thereby converting aster-shaped anisotropies into net forces and torques that move and orient centrosomes. Aster shapes may, in turn, be regulated by cell shape, aster-to-aster interactions, or the presence of asymmetric yolk layers (figure 11(A), [104]). These mechano-geometric mechanisms provide a simple, yet powerful iterative design that accounts for many features of early embryos, such as the typical orthogonal sequences of early blastomere divisions (figure 11(B), [103]).

Although modelling and cell manipulation support these models [101-103], it is still the case that little is understood about how dynein may pull in bulk and organize aster forces. Addressing such mechanisms constitutes one important challenge. Dynein could, for instance, pull on static or elastic structures in bulk, such as networks of intermediate filaments or actin. Alternatively, dynein could exert bulk forces from the viscous drag of endomembranes and vesicle cargos it transports to the aster centre [105]. Tests of these hypotheses will necessitate the manipulation of bulk elements, the proper measurement of flows at multiple scales, as well as the development of modelling frameworks to understand how force-generating events are integrated to produce the measured net forces and torques applied by MT asters [106]. A second feature of this view is that asters must stop growing in the vicinity of the cortex, yolk layers, or neighbouring asters. The dynamic regulation of plus-end MT growth, through motors, bundlers, end-binding, or depolymerizing factors, is clearly involved, but the mechanisms are still partially lacking [104]. Finally, large asters that span hundreds of micrometers depart from the standard radial-organization picture and feature bushy, branched and poorly connected MTs. The way in which asters organize and grow and how forces are eventually transmitted to the centrosomes are other fascinating problems to solve in the future [104].

#### 12.2.2. The diversity of cleavage patterns

Cleavage patterns are regular and often invariant among large numbers of species, indicating a high level of regulation. Yet, these divisions can reorganize in response to physical manipulations such as e.g. egg bisection, centrifugation or shape modulation, suggesting that they rely more on self-organization than deterministic programs. Furthermore, early embryos loaded with maternal material develop with a near-constant cytoplasm biochemistry [100]. These considerations raise the question of how and where deterministic cues may compete or cooperate with self-organizing designs such as those based on aster shape and length-dependent forces (figure 11). Many embryos inherit maternal polar domains that prime cortical dynein pulling for aster de-centralisation and asymmetric division. Yolk layers may also constitute an initial spatial cue that organises aster position and cell division [99, 103]. Although current models typically implement those guiding cues as fixed initial conditions [103], those cues most likely mature in strength or even change position through their interactions with growing and moving asters. Fertilizing embryos also feature large-scale acto-myosin driven flows that may move yolk, cortical domains or endomembranes. Finally, asters may also contribute to control surface mechanics by spatially regulating cell-to-cell adhesion and contractility. Thus, one exciting possibility is that both the robustness of embryonic cleavage, as well as their diversification, may result from the tuning of different feedback loops, rather than from pre-determined genetic information. The challenge ahead is thus mostly embedded in documenting and studying these interplays, and how they may predict the morphological features of multiple embryos. The use of emerging synthetic methodologies to move the division apparatus, create flows or displace cortical domains will be instrumental in achieving this future challenge [106].

#### 12.2.3. Relevance to smaller cells in tissues

When studying cell division in small somatic cells, what can we learn from the divisions of large eggs and blastomeres? Small cells are likely to be dominated by surface effects, and centrosome positions and cell division may rather hinge on cortical MT forces and dynein activity. However mechanisms for dynein pulling implicating endomembranes and bulk hydrodynamics are likely to be relevant to small cells as well, because their internal organization and cytoplasm physical properties are similar to those of large eggs and blastomeres. In spite of plausible divergent regulatory mechanisms, adherent and tissue cells also respond to geometric cues and usually orient their division along the long cell-shape axis [98]. Remarkably, recent findings even suggest that the large-scale architecture of developing epithelial tissues (e.g. squamous, cuboidal or columnar) may also depend on iterative shape-division interplays [107]. Addressing the titration of these interplays by effectors of surface mechanics and division positioning certainly constitutes another challenge for future studies addressing the contribution of proliferation to tissue dynamics.

### 12.3. Advances in science and technology to meet challenges

One aspect which has limited studies of embryonic cleavages is the accurate dynamic 3D imaging of blastomere shapes, internal organization, and division. There is thus a need to implement methods enabling high-resolution 3D live imaging in large and poorly transparent embryos. Light-sheet, multi-photon microscopy, and advances in electron tomography are promising avenues. In addition, modelling approaches are thus far restricted to mechanical inputs, the identification of equilibrium division positions and orientations, and cell shapes [98, 106, 107]. The implementation of novel simulation platforms, integrating cytoskeleton dynamics such as aster growth, rearrangements, endomembranes and hydrodynamic interactions, will be instrumental to derive comprehensive mechanisms. The study of the spatial organization and division of early embryos could serve to motivate the development of these technologies.

### 12.4. Concluding remarks

As discussed throughout this text, studies in the past decade have revived the early interest in studying cleavage patterns, not only from a developmental perspective, but also as a means to understand cell division and morphogenesis as a whole. To date, many patterns still remain mostly enigmatic. Spiral cleavage, for instance, is a highly represented pattern in invertebrates, for which basic documentation and mechanisms are still lacking. The re-emergence of non-model systems in modern biology will certainly help to fill this gap and address how evolution has tuned specific biological and physical parameters to create the astonishing diversity of cleavage divisions and early embryo morphogeneses.

## 13. Building a mechanical atlas of the preimplantation embryo

### 13.1. Status

During preimplantation development, the mammalian embryo forms a blastocyst, which will implant into the maternal uterus. The blastocyst is initially composed of two distinct lineages: the TE and the inner cell mass (ICM). The TE, which envelops the ICM and a fluid-filled lumen called the blastocoel, is responsible for invading the uterus and contributes to placental tissues. On the other hand, the ICM gives rise to all embryonic tissues as well as extraembryonic derivatives. Decades of research revealed how the architecture of the blastocyst is crucial for the differentiation of the first lineages and for defining the first axes of the mammalian body. However, it is only recently that studies have focussed on the question of what shapes the architecture of the blastocyst.

The shaping of the blastocyst can be broken down into distinct morphogenetic steps, that occur as cells undergo synchronous cleavage divisions. In the mouse, these take place in the following sequence: compaction, internalization, and lumen formation at the 8-, 16- and 32-cell stages, respectively. Compaction consists of a developmentally regulated wetting process, during which, cells are drawn into close contact. During internalization, the precursors of the ICM envelop themselves with the precursors of the TE until they are no longer in contact with the outside medium. Lumen formation results from the accumulation of fluid within the intercellular space, which separates contacts between the ICM and some of the TE cells. This morphogenesis takes place within the zona pellucida, a porous elastic capsule made of glycoprotein, from which the embryo hatches before implantation.

Mechanical changes driving the morphogenesis of the blastocyst have already been identified using several methods, including laser ablation and micropipette aspiration. Such methods can exploit key advantages provided by this experimental system: a countable number of large cells that divide and develop slowly enough to perform repeated measurements. This makes the preimplantation embryo an ideal platform to characterise cell and tissue mechanics extensively, to understand embryo morphogenesis, and to compare different methods of mechanical measurements.

### 13.2. Current and future challenges

Several methods have been used to characterise the mechanics of the developing blastocyst: laser ablation, micropipette aspiration, AFM and deformable hydrogels. These methods pose distinct challenges in terms of invasiveness (access and damage to the sample) and in the type of measurement they offer (absolute values in physical units, information about anisotropy, different model assumptions). This roadmap delineates the methods and associated challenges that can be used to build a mechanical atlas of the developing preimplantation embryo.

Laser ablation offers the unique possibility to probe tension anisotropies from the sub-cellular to the tissue scale. After the laser ablation of pre-stressed materials, a recoil can be observed and its initial velocity is proportional to the stress and friction within the material. Therefore, measurements of the recoil velocity can be used to infer tension anisotropies *in vivo*, provided that friction within the material is unchanged. During the internalization process that will form the ICM, laser ablation has revealed tension anisotropies with larger tensions directed towards the internalizing cell [108]. This is caused by higher tension in the internalising cell itself [109]. Laser ablation was also used to assess the tension at tight junctions before and after the zippering of a ring of actin surrounding the apical domain [8]. However, it remains unclear whether the measured differences in recoil velocities were due to changes in tension within the actomyosin cytoskeleton or to changes in friction within the maturing cell-to-cell contacts. One final cautionary note: although laser ablation provides a very practical way to peer into sub-cellular forces, it can be misleading if uncontrolled, as severe damage, for example, that causing cell fusion [110], can also affect the recoil. It can also be difficult to determine what is being ablated and, for example, an attempt at cutting a filopodia at the cell surface can also sever the nearby actomyosin cortex [110].

Micropipette aspiration can be used to exert controlled pressure from sub-cellular to tissue scales, and surface tensions can be calculated using the Young–Laplace equation. This assumes that the sample behaves as a liquid droplet, whose curvature depends on the ratio between its pressure and surface tension. Unlike laser ablation, micropipette aspiration is a contact method, which requires stripping the embryo from its zona pellucida. Applied throughout the 8-cell stage, micropipette aspiration has allowed a spatio-temporal map of surface tension to be built at the sub-cellular level and using absolute values [111]. Measuring absolute values was critical to evaluate the relative contributions of the tension changes at cell-to-medium versus cell-to-cell interfaces. This revealed that three quarters of compaction is driven by an increase in surface tension at the surface of the embryo and one quarter by the relaxation of cell-to-cell contacts. Furthermore, because micropipette aspiration does not damage the sample, it allowed the evaluation of the tensions of cells before they internalized to become part of the ICM during the 16-cell stage. This was essential to follow cells and test a theoretical prediction that cell sorting would occur if and only if the tension ratio between neighbouring cells grew above 1.5 [109]. Later, at the blastocyst stage, after blastocoel expansion, micropipette aspiration experiments have reported different surface tensions between the TE lining the ICM and the TE facing the blastocoel [112]. The biological significance of this difference remains unclear and will require further investigation. Also, at this stage, cells have become smaller with cleavage divisions, they stretch extensively, and the nucleus becomes too bulky not to be aspirated into the micropipette. This makes the liquid droplet assumption difficult to justify and the interpretation of micropipette aspiration delicate [112].

In addition to micropipette aspiration, the properties of the TE were also probed using AFM, a rapid and very sensitive method for measuring the mechanical properties of living materials [112]. AFM records the bending of a cantilever while pressing on the embryo, which, in this experiment, had been stripped from its zona pellucida and glued onto a culture dish. The AFM measurement was done by compressing whole blastocysts and the apparent elasticity of the TE was extracted using an elastic shell model. The validity of this model was not tested and it remains unclear how much mechanical resistance to compression is provided by the epithelial shell and by the lumen that it envelops.

Multiple techniques have been used to evaluate the pressure of the lumen, and these can be directly compared. Introducing a needle into the blastocoel of an intact embryo and equilibrating the fluid flow allowed the hydrostatic pressure inside the blastocoel to be measured at ~1 kPa [112]. However, the rapid loss of epithelial integrity and rapid pressure drops limit the timescale of this measurement to a few seconds. Less-invasive micropipette aspiration at the embryo surface was used to measure pressure while following normal development. Using the Young–Laplace equation yielded the hydrostatic pressure of the blastocoel as ~300 Pa [113]. However, similarly to the AFM approach mentioned above, this measurement was not able to distinguish between the contributions of the blastocoel and the overlying epithelium. Also, micropipette aspiration and AFM measurements were performed on embryos without the zona pellucida. Alternatively, placing embryos within their zona pellucida into hydrogels of known stiffness allowed the gel deformation to be monitored upon blastocoel inflation. This revealed blastocoel pressures of ~1.5 kPa and a key aspect regarding the pressure of the blastocoel: building up pressure above a few hundreds of Pa requires the presence of the elastic confinement of the zona pellucida [114]. This method and the associated model also allowed the measurement of a battery of biophysical properties of the blastocyst, such as the elasticity of the zona pellucida and the permeability of the TE to water and osmolytes. Such data will be key for our understanding of blastocoel formation and maintenance.

### 13.3. Advances in science and technology to meet challenges

Surface tension and pressure measurements in the forming blastocyst have established the preimplantation embryo as an ideal system to study the mechanics of embryonic development. Looking forward, it is now possible to apply additional techniques to extensively characterise the biophysical properties of the mouse embryo and build a mechanical atlas of preimplantation development. Such an atlas will constitute a precious blueprint for future studies. On the one hand, it will facilitate comparison and benchmarking techniques as applied to mouse embryos [115]. On the other hand, it will facilitate the comparison of mechanical properties across different tissues and species. In addition, it will provide material for theoreticians to build and test increasingly faithful models of preimplantation development. For this to be possible, building the atlas requires rigorous and ambitious standards: absolute measurements in physical units.

Additional mechanical properties can readily be measured with current technologies. For example, cell and tissue viscosity can be assessed using micropipette aspiration. The mechanical stability of cell-to-cell contacts can be measured using a dual pipette assay or AFM. The mechanical properties of the plasma membrane could be characterized using AFM, optical or magnetic tweezers. These will provide unique insights into the role of these properties in preimplantation development.

Other biophysical properties will require the development of new tools. In particular, the role of intercellular fluid in shaping tissues will benefit from the biophysical measurement of cells’ and junctions’ permeabilities to different solutes. This would allow a precise assessment of their relative contribution to osmotic pressure. In addition, understanding the contribution of nuclear mechanics to the deformation of the embryo at later stages of preimplantation development, when nuclei deform significantly, will require rigorous measurements and theoretical interpretations.

### 13.4. Concluding remarks

Within five years, preimplantation development went from being mostly uncharted to one of the best mechanically characterized experimental systems for studying morphogenesis. This allows us to envision the construction of a mechanical atlas of preimplantation development in the near future. Such an atlas will be a unique tool for comparing biophysical measurements to benchmark techniques and to understand more precisely the molecular and cellular origin of the emerging biophysical properties that are measured. This atlas of mechanical properties in absolute values will also allow a direct comparison of preimplantation development in different mammalian species, such as humans, and thereby open up a new field of research: evolutionary mechanics.

## 14. Physical principles of the organization of the cell cycle and morphogenesis in early *Drosophila* embryos

### 14.1. Status

**Figure 12.**
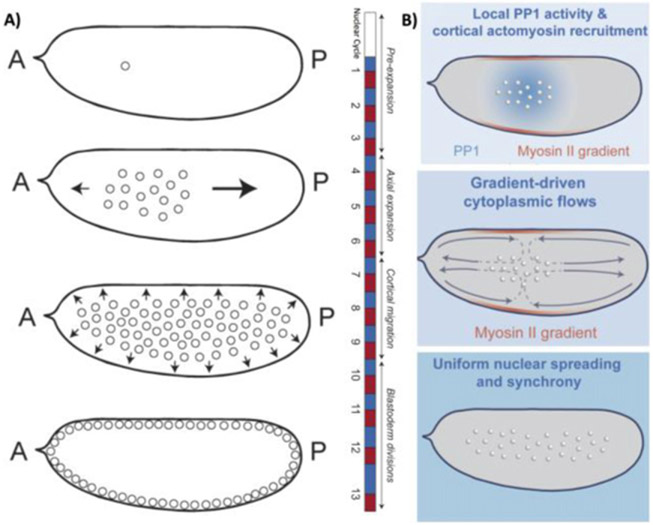
Stages and mechanisms of control of cleavage divisions in early embryogenesis of *Drosophila*. (A) Nuclear-cycle stages. Following fertilization, the nuclear cycles are characterized by stereotypical movements: axial expansion denotes the movement of nuclei along the AP axis (cycles 4–6); cortical migration denotes the movement of nuclei to the cortex (cycle 7–9). (B) Model for axial expansion as in proposed in [120].

Like the embryos of many other species that develop outside the mother, *Drosophila* embryos are large. Following fertilization, they undergo a series of rapid cleavage divisions to increase DNA content prior to gastrulation [116]. In these early stages, the embryo is a syncytium, thus the rapid nuclear divisions generate a large multinucleated cell. Following a cycle of 13 precisely timed and reproducible rounds of division (figure 12(A)), the embryo finally undergoes the maternal-to-zygotic transition. At this transition, zygotic gene expression increases, the cell cycle slows down, a specialized form of cytokinesis encloses the nuclei into membranes—a process known as cellularization—and gastrulation begins [117].

To ensure that these processes occur in a coordinated manner, it is important that the cell cycle is synchronous across the embryo during in the early cycles. It has recently emerged that the synchrony of these divisions requires specialized processes [118-120]. Three of the early nuclear cycles (nuclear cycles 4–6) are characterized by rapid nuclear movements, which ensure that nuclei travel along the anterior–posterior (AP) axis to uniformly fill the embryo (figure 12(A)). These movements are instructed by the cell-cycle machinery, yielding an interesting example of integration of biochemical and mechanical signals [120] (figure 12(B)). In brief, the activity of one of the major kinases driving the cell cycle—the cyclin-dependent kinase 1 (Cdk1)—undergoes oscillations restricted to a small region of space surrounding the nuclei. The local decline in Cdk1 activity at mitotic exit triggers the activation of mitotic phosphatase PP1 within a region of space comparable to the distance between the nuclei and the cortex (40–50 microns). Thus, at the end of mitosis/early interphase, PP1 is specifically active in a region of the cortex overlying the nuclei. Locally active PP1 can trigger the cortical activity of the GTPase Rho, which in turn triggers myosin activation. When myosin activation is not uniform across the cortex, the force imbalances generated by tension gradients induce embryo-wide cytoplasmic flows that have the correct geometry to move the nuclei so as to fill the entire embryo (figure 12(B)). Once the nuclei are uniformly placed, the coupling of cell cycle machinery and actomyosin contractility remains active, but the uniformity of myosin at the cortex precludes the generation of further flows. Thus, the mechanisms that drive proper nuclear positioning in the early *Drosophila* embryo arise from the integration of biochemical (cell-cycle oscillator) and mechanical signals (actomyosin contractility and hydrodynamic flows).

Following the process of axial expansion, nuclei migrate towards the embryo’s surface during cell cycles 7–9 to generate a blastoderm of uniformly spaced nuclei. At the surface, the embryos undergo 4 additional rounds of meta-synchronous divisions. These divisions do not occur at the same time across the embryo but display wave-like patterns. *In vivo* measurements of Cdk1 activity, mathematical modelling and theoretical analyses have demonstrated that the mitotic waves are controlled by waves of Cdk1 activity [118, 119]. It was demonstrated that these waves do not arise through trigger waves, as previously described. Although the Cdk1 regulatory system exhibits bistable dynamics in *Drosophila* embryos, during the very fast early cell cycles, the rapid drive that moves the system out of a bistable regime outpaces the state switching that underlies propagation of a trigger wave. In this scenario, waves arise from the following mechanism. The early dynamic and spatial coupling of Cdk1 activity generates activity gradients. As the cell cycle progresses, bistability is lost almost simultaneously across the embryo. When this happens, the activity of Cdk1 increases at a uniform rate across the entire embryo, which results in a wave-like pattern of mitotic entry. Following this stage, clock-like processes ensure that a wave of mitotic exit follows the wave of mitotic entry [118, 119]. Thus, the synchronization of the cell cycle prior to the maternal-to-zygotic transition arises from a reaction–diffusion mechanism generating a wave-like spread of activity in a transiently bistable system [119]. The recent progress described above on the understanding of the cleavage divisions of *Drosophila* embryos highlights the potential of this system to uncover novel physical principles of morphogenesis.

### 14.2. Current and future challenges

Despite much recent progress, several important questions remain open in the field. Novel insights will also be gained by comparing cleavage divisions in *Drosophila* to those in other species. For example, a recent study of the coupling of the cell cycle and actomyosin dynamics in zebrafish embryos reveals how this interplay controls the separation of yolk and cytoplasm [121]. In this system, ooplasmic segregation is not driven by cortical contraction, but rather by the contractility of bulk cytoplasmic actomyosin. Consistently with previous findings [122], bulk actomyosin contractility is stimulated and not inhibited by Cdk1 activity. The mechanisms and full implications of this different regulation of actomyosin contractility by the cell cycle (stimulated in the bulk and inhibited at the cortex) remain to be explored. Furthermore, ooplasmic segregation arises from the opposite movements of yolk and cytoplasm, which are controlled by a wave of actin assembly that pulls cytoplasm and pushes yolk granules. An understanding of the hydrodynamic coupling of actomyosin gels and the cytosol (the soluble part of the cytoplasm), and how they can be modulated to generate flows of geometric features desired for different functions, remains a fundamental open question in morphogenesis [123]. Moreover, the study of these flows offers a unique perspective from which to gain insights about the physical properties of cytoplasm and to dissect under which conditions the cytoplasm behaves as a simple fluid or a complex one.

Insect embryos vary in size and shape, but the way that the coupling of cell cycle, cortical contractility, and cytoplasmic flows are influenced by geometry and how they scale with size remains to be explored. The positioning of nuclei does not only rely on actomyosin-driven flows. Following axial expansion, nuclei migrate to the cortex through an MT-driven process. Whether this process involves a similar integration of cell cycle and cytoskeletal dynamics remains to be elucidated. Moreover, it is likely that not only nuclei but many other organelles or important molecules (for example morphogens) are transported by cytoplasmic flows.

Recent work in both *Drosophila* and other systems [124] has demonstrated the importance of travelling waves of signalling activity as a mechanism for coordinating biochemical dynamics across large scales. It will be interesting to study the ability of chemical waves to propagate through complex multicellular tissues using extracellular ligands or other means of inter-cellular communication.

### 14.3. Advances in science and technology to meet challenges

The recent progress in understanding the cleavage divisions of *Drosophila* embryos was made possible by the use of a biosensor for cell-cycle dynamics (a fluorescence resonance-energy-transfer biosensor revealing the ratio of activities of Cdk1 and PP1). Moreover, important and definitive insights into the connection between cell-cycle oscillations and cortical actomyosin dynamics were established using an optogenetic method to control cortical contractility [120]. We expect that further development of biosensors and optogenetic methods will afford the field a unique ability to monitor and manipulate biochemical and mechanical signals in morphogenesis. These methods could be complemented by physical manipulations to alter cytoplasmic flows, for example, by generating artificial temperature differences inside the embryo [125].

The analyses of cytoplasmic flows have been mainly limited to two-dimensional projections. Recent advancements in light-sheet imaging suggest that it might be possible in the future to perform a full three-dimensional reconstruction of cytoplasmic flows.

### 14.4. Concluding remarks

Recent quantitative work in *Drosophila* embryos has revealed novel concepts for the spatiotemporal coordination of the cell cycle during rapid cleavage divisions. These experimental and theoretical results have important parallels and analogies to many other embryonic systems, suggesting shared physical principles for the organization of embryogenesis. Dissecting the generality of these mechanisms and their extension to more complex adult tissues has the potential to provide a new quantitative paradigm for developmental biology.

## 15. Dynamic interplay of cell-contact geometry and signalling

### 15.1. Status

In recent years, it has been increasingly evident that cellular and tissue morphology are tightly regulated to achieve specific cellular organization in terms of the number of cells, their types, their positions, and their sizes. Such cellular organization requires feedback from cellular morphology to control differentiation, cell movement, cell death, and more. For example, during epithelial growth in Drosophila wing disks, cell competition drives specific cells to go through apoptosis, in a process that depends on the number of neighbours expressing high *myc* levels [126].

This example and others raise the question of how morphological information is converted into biochemical signals that regulate differentiation. An appealing possibility is that changes in cellular morphology affect cell-to-cell signalling through properties of the contacts between cells. Several recent works have discussed this possibility in the context of Notch signalling, which requires direct cell-to-cell contact between the sender and receiver cells and hence is likely to be affected by contact morphology.

**Figure 13.**
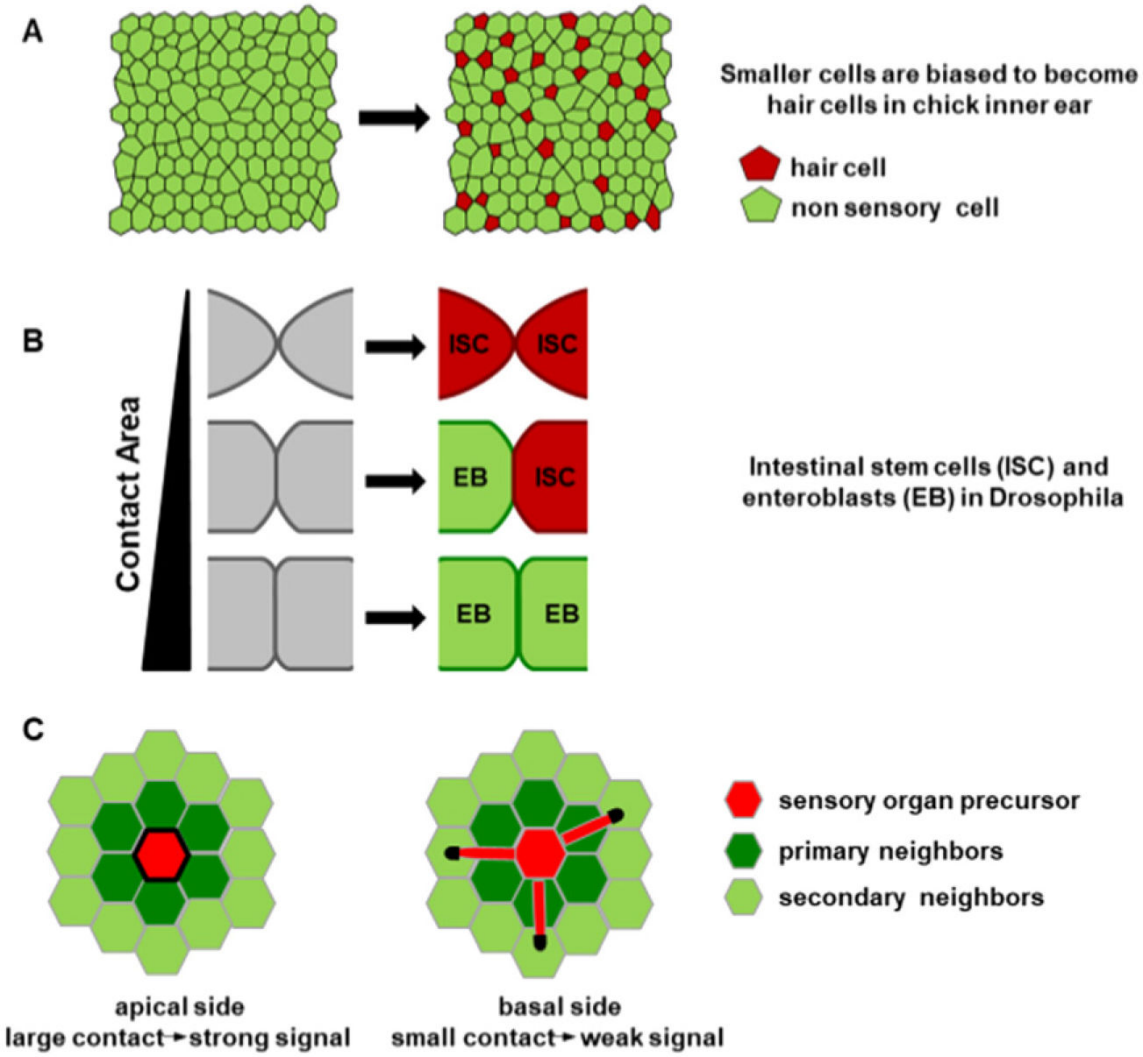
Examples where contact morphology affects cell fate. (A) In a chick’s inner ear, smaller cells are more likely to become hair cells [127]. (B) Fate switch in the Drosophila intestine depends on contact area [128]. (C) Signalling level is weaker for cells contacting through basal filopodia compared with cells in direct apical contact in Drosophila bristle patterning [129].

Shaya *et al* have shown that the dependence of Notch signalling on contact area can explain why smaller cells are more likely to differentiate into hair cells a during chick’s inner-ear development [127] (figure 13(a)). Guisoni *et al* showed that the Notch signalling strength between pairs of intestinal stem cells correlates with their contact area and influences their differentiation into different intestinal cell types [128] (figure 13(b)). Finally, Hunter *et al* showed that Notch signalling through basal filopodia contacts is weaker than via the larger apical contacts during the differentiation of sensory organ progenitors in flies [129] (figure 13(c)).

### 15.2. Current and future challenges

The relation between contact morphology and signalling poses several significant challenges. Establishing a causal relation between contact morphology, contact time, and signalling level is difficult because of several factors. First, it is unclear how signalling can be uncoupled from different sources. Cells are typically surrounded by multiple neighbouring cells and receive multiple signals.

**Figure 14.**
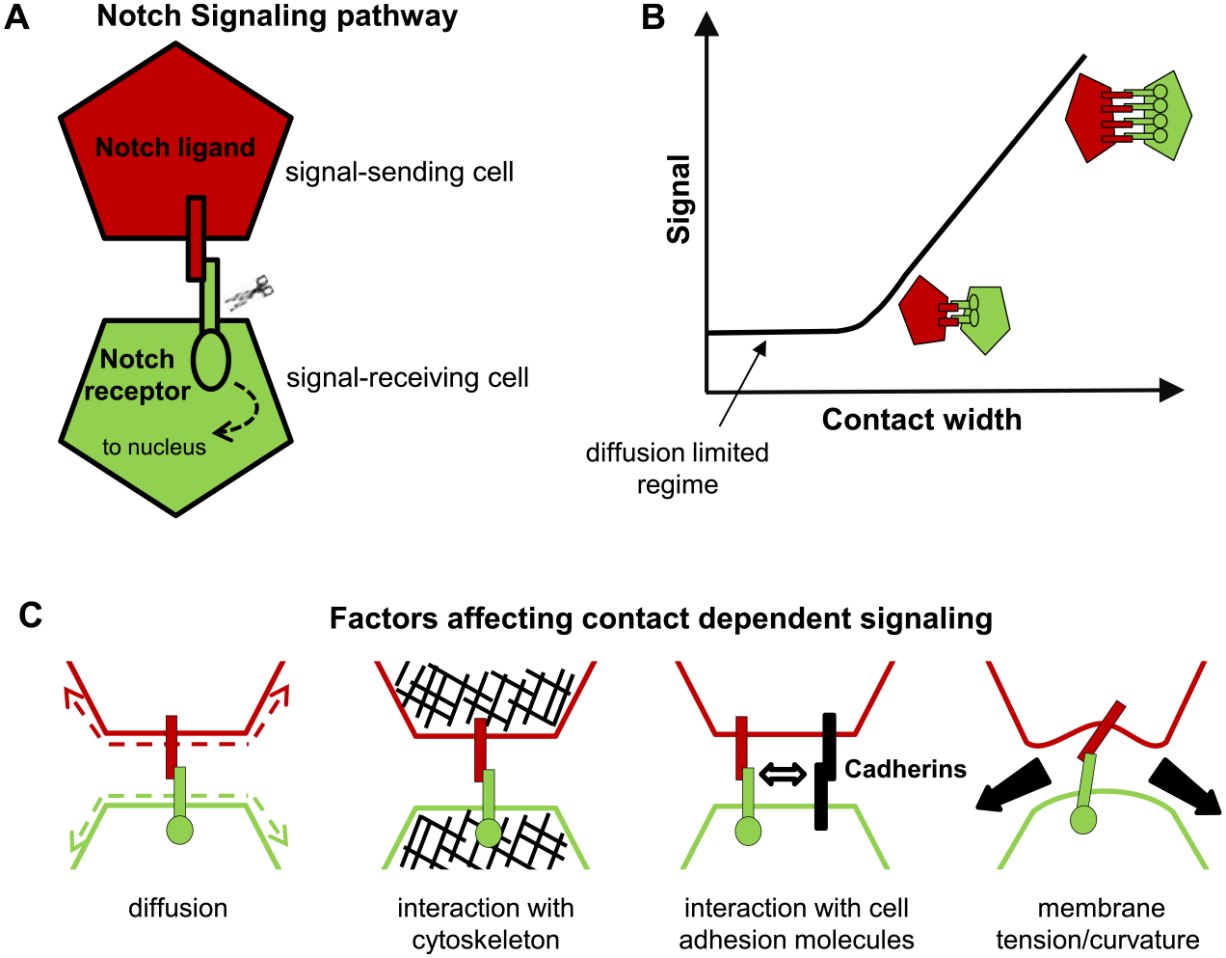
Dependence of Notch signalling on contact area. (A) Schematic of Notch signalling. (B) Dependence of Notch signalling on contact width. Diffusion-limited regime occurs when the contact width is smaller than the diffusion-length scale. (C) Schematic of factors affecting contact dependence of signalling.

This can be solved, in some cases, by looking at situations where communication is restricted to pairs of cells. Examples of such situations *in vivo* can be found during asymmetric cell divisions such as those occurring in the intestinal stem cells mentioned above [128] and in the sensory organ precursor in flies [130]. In these cases, signalling can be correlated with the contact area, however, it is often hard to manipulate the contact area to show the direct effects of contact on signalling. *In vitro* systems may offer better control of the contact area between cells. Shaya *et al* used a double micro-well to measure Notch signalling between a sender and a receiver cell, where the contact width was able to be controlled by the width of the neck connecting the two wells [127]. Their measurements showed that Notch signalling correlated with the contact width, for contact widths ranging from 1–40 *μ*m (Figures 14(a) and (b)).

Another factor is the difficulty of associating the intracellular signalling response with events that occur at a specific cellular interface. In a recent elegant work, Trylinski *et al* [130] used Notch tagged with a photoconvertible fluorescent protein to show that signalling between pIIa and pIIb daughter cells during asymmetric cell division of the sensory organ precursor in Drosophila originates from a specific sub-apical compartment between the cells. This was done by converting only the Notch molecules in this sub-apical compartment to red fluorescence, and showing that these converted Notch molecules are the ones that accumulate in the nucleus.

Finally, fast signalling readout is needed to allow tracking of the dynamic relation between contact morphology and the signalling response. Standard transcriptional reporters are often too slow to track dynamic signalling, as they often respond within a timescale of tens of minutes to hours. Methods that track faster signalling events include tracking the translocation of NICD to the nucleus [130] and tracking mRNA production in active transcription loci using MS2-GFP [131].

A second challenge is to develop methods to identify and characterize the molecular and morphological properties of the contact area, beyond the contact area itself, that affect signalling. These include the membrane diffusion rates of the receptors and ligands at and around the contact site, interaction with cell-adhesion molecules such as cadherins, interaction with the cytoskeleton, and membrane tension and curvature (figure 14(c)). For Notch signalling, several recent works provide initial clues as to how these properties affect signalling. For example, the diffusion rates of Notch receptors and ligands were shown to be important in determining whether Notch signalling would exhibit contact dependence [132]. For contact areas smaller than the membrane-diffusion length scale, it is expected that signalling would no longer depend on the contact area (see figure 14(b), diffusion-limited regime). This property can potentially explain why signalling mediated by filopodia, where the contact area is very small (of the order of ~0.1 *μ*m in diameter), can still produce significant signalling output.

The interaction with the cytoskeleton can also significantly affect contact-dependent signalling. For example, it was recently shown that activation of the actin nucleator Arp2/3 is required for the activation of Notch in certain contexts, but not in others. It has been suggested that Arp2/3 mediated nucleation of actin is important for endocytosis of the Notch ligand which is required during Notch activation [133]. An appealing hypothesis associated with this observation is that membrane tension at the cell boundary may affect Notch activation, however, direct evidence for the effect of tension on Notch signalling is still missing.

A third challenge is to understand how feedback between signalling and morphology underlies developmental patterning processes. Many studies focus either on the molecular regulatory level, ignoring cell morphology, or focus on changes in cell morphology as a downstream consequence of the cell-fate decision processes. Yet, it seems that in many developmental processes, cell-fate decisions occur concurrently with morphological changes. We therefore need to develop methods for experimentally identifying such feedback, and to theoretically model the developmental processes that rely on such feedback. An example of such feedback between signalling and contact properties was recently discussed in the context of Nodal signalling [134]. In that system, positive feedback between contact duration and Nodal signalling controlled a cell-fate switch during zebrafish gastrulation.

### 15.3. Advances in science and technology to meet challenges

To address these challenges, we need to develop tools that can enable better spatiotemporal control and readout of signalling. A promising direction is to employ optogenetic tools to spatially and temporally induce signalling, such as those developed for controlling membrane channels and the cytoskeleton. Light-induced signalling will allow local signalling induction at specific cellular interfaces, both *in vitro* and *in vivo*. Furthermore, optogenetic tools may also allow control of the geometry of the cell, by regulating local actomyosin activity. Initial steps towards this goal were recently reported, where optogenetic inhibition of Delta in flies was used to study dynamic signalling responses [135]. One challenge with the currently available optogenetic tools is to be able to use these tools and, at the same time, image the response using fluorescence microscopy. Current optogenetic tools are activated by a broad spectrum of blue/green light (LOV-domain-based systems) or by red/far-red light (phytochrome-based systems) thus limiting the ability to image multiple fluorescent protein reporters. Therefore, more versatile light-inducible systems that could allow the spatiotemporal control of signalling and the use of the full spectrum of fluorescent reporters for imaging, would be extremely useful.

On the theoretical side, we also need to develop modelling and computational tools that will allow study of the feedback between cell morphology and cell-to-cell signalling. In recent years, 2D vertex mechanical models have successfully been used to understand how cell and tissue morphology are determined by the mechanical forces acting on each cell and each boundary. These models would have to be expanded to include the regulatory and signalling states of cells, typically described by rate equations. In such ‘hybrid’ models, the regulatory state of the cell (e.g., the level of signal or concentration of transcription factor) can affect the mechanical properties of the cell, while the morphology of the cells, determined by mechanical forces, will affect cell-to-cell signalling. Generating such models in a way that will be accessible to the community will require the development of new modelling and computational platforms that bridge the gap between these two modelling modalities.

### 15.4. Concluding remarks

The study of the interplay between contact morphology and signalling is still in its infancy. We expect that as new advances in technology and modelling emerge, we will gain further insights into the role of this interplay in various developmental systems. This interplay will most likely be expanded to additional signalling pathways, both at short and long range.

## 16. From locally generated forces to global shape and back

### 16.1. Status

The way in which the shapes of organs and organisms emerge from the interaction of cells has been a central question of the past decades. This question has mainly been formulated with the strong assumption that cells are endowed with physical properties and rules of interactions that guide how they arrange and grow to form functional structures. This view often obscures the fact that morphogenesis in animals and plants is the process by which tissues, organs and organisms acquire their shape given boundary conditions that impose geometric, mechanical and biochemical constraints. Recent works have started to emphasize the importance of tissue boundaries and their role in morphogenesis. One prominent example is the extension of the *Drosophila* embryo germband: in this tissue, local forces generated by actomyosin contractile networks remodel cell contacts to produce anisotropic cell rearrangements. While this process occurs across the germband, an external tissue, the forming endoderm, pulls on the germband, thereby guiding its extension [136, 137].

**Figure 15.**
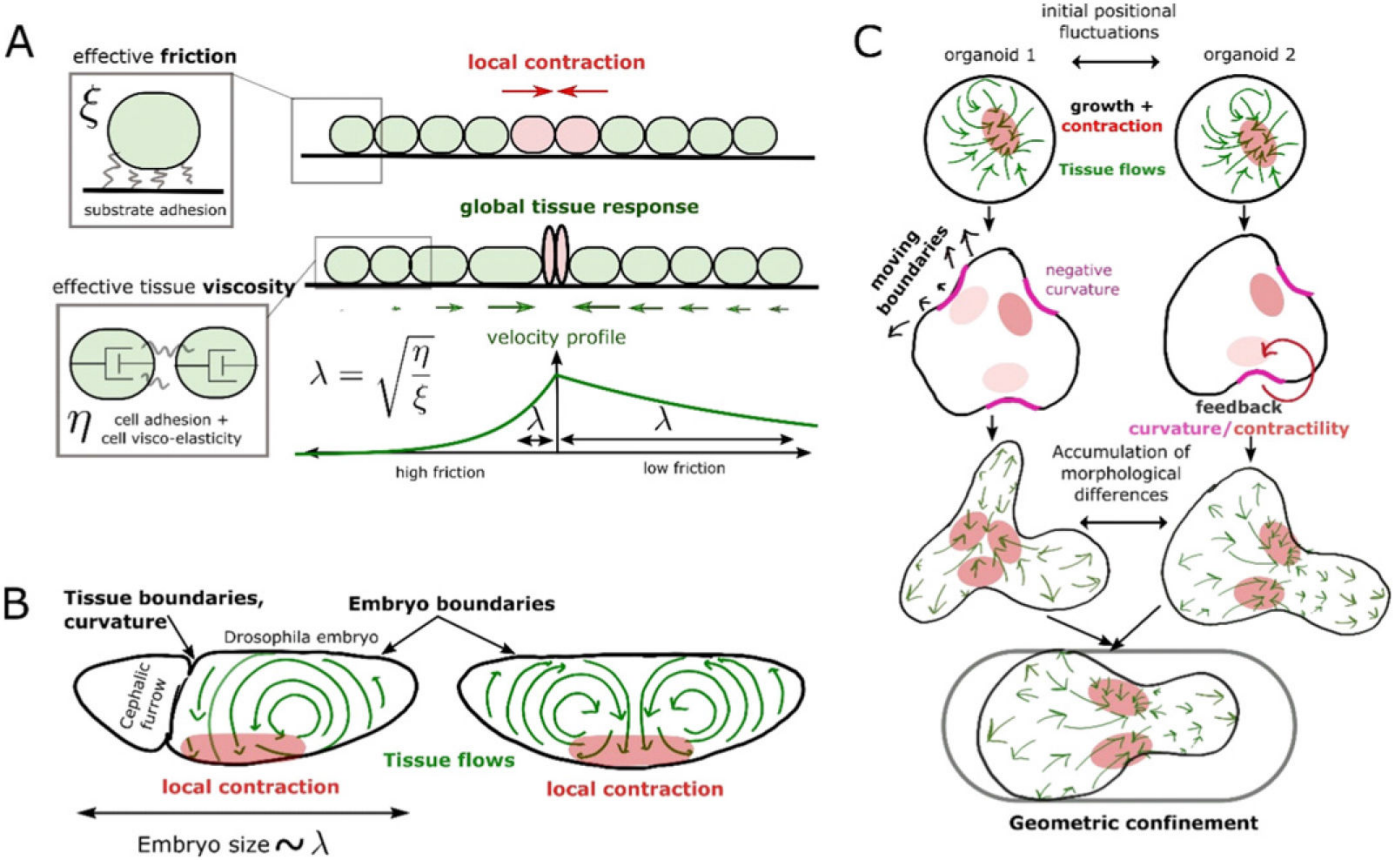
(A) Local tissue contractions can produce deformation/tissue flows over a length scale *λ* which is dependent on material properties. In the case of a viscous tissue in interaction with an external substrate, this length scale varies with tissue viscosity and the effective friction coefficient between the tissue and the substrate. (B) Geometric boundaries guide flow patterns. Simulations of tissue flows during Drosophila morphogenesis [138] demonstrate how a local tissue contraction in the germband can generate a global tissue flow whose geometry is influenced by the embryo’s geometry. In the absence of the cephalic furrow, which is a highly curved interface separating the anterior part and the posterior part of the embryo, the global tissue flow is highly affected. (C) Coupling between the tissue’s moving boundaries and bulk tissue flows in an organoid system. Mechanisms coupling the tissue boundary geometry (here, its curvature) with the generation of local active stresses can generate instabilities leading to the shape variability observed in organoid systems. Local mechanics and global geometry are coupled to give rise to shape: this coupling could be perturbed by imposing geometrical constraints on the organoid using micro-patterning, for example.

This example and others point to the necessity of considering morphogenesis as both a local and a global problem. In closed systems with fixed external boundaries such as the Drosophila embryo, mechanical models inspired by hydrodynamic theory have shown that local mechanical stresses, together with geometric constraints, lead to macroscopic flows that pattern the embryo [138, 139]. The extent to which locally produced deformations affect or are affected by boundaries depends on the geometric and material properties. For viscous materials subject to external friction, one can define a hydrodynamic length that describes the length over which the effects of a local force are felt (figure 15(A)).

When the hydrodynamic length is of the same order as the embryo size, the boundary conditions strongly affect the pattern of tissue flows. Boundaries are usually defined by an external envelope (in the case of the embryo) or neighbouring tissues (in the case of organs). For instance, the presence of a cephalic furrow (characterized by a local fold of the tissue separating the head from the tail) in the *Drosophila* embryo acts as a barrier to tissue deformation (figure 15(B)).

Understanding the origins of these flows requires the local and global scales to be disentangled, and therefore requires the ability to independently perturb the locally generated forces and the global geometric constraints to which the tissue is subjected. In model systems such as Drosophila, local forces can be controlled by acute genetic perturbations, optogenetic approaches, and direct optical manipulation [140]. The spatiotemporal variations of tissue mechanical properties can be experimentally measured *in vivo* with magnetic and optical manipulation [141].

It is also possible to create artificial boundary conditions by laser cauterization [136] to mechanically uncouple the interactions of different tissues and understand their respective contributions to flows and deformation.

### 16.2. Current and future challenges

Challenges in this field are both conceptual and technological. Current studies mainly address the two-dimensional dynamics of epithelial tissues in contact with rigid boundaries. The current challenge is to extend these approaches into three dimensions and to develop new concepts to study systems whose global geometry is not fixed (deformable boundary conditions). How do 3D tissue flows within an organ shape its boundaries? In turn, how do these deformable boundaries affect the internal flows?

Tackling these questions requires a strong dialogue between experimental and theoretical approaches. Ideally, the experimental system should be amenable to perturbations that affect boundary conditions, system size and system geometry. There have been successful attempts, using this approach, to perturb boundary conditions *in vivo* but this remains limited to a few examples, and proves to be extremely difficult in mammals due to their lack of accessibility post-implantation.

In parallel, the emergent study of embryonic organoids has revealed the capacity of *in vitro* multicellular systems to self-organize into structures mimicking organs and parts of embryos. For instance, mouse embryonic stem cells are able to assemble 3D aggregates, dubbed gastruloids, that undergo gastrulation-like events and elongate. They are able to organize a post-occipital pattern of neural, mesodermal and endodermal derivatives, recapitulating the embryonic spatiotemporal pattern of gene expression [142]. They can be prepared in controlled conditions (size, confinement geometry, matrix stiffness, chemical environment). The effect of fixed boundary conditions has been proven to play a key role in the gene patterning of 2D gastruloids confined on micropatterns (see Section 2). In contrast, the boundaries of 3D gastruloids can move freely, and their shapes evolve through a combination of growth and elongation. The variability in shape and patterning of gastruloids (for a fixed initial number of cells) suggests that they lack certain external mechanical constraints that are present in embryos and that are critical to robust morphogenesis (figure 15(C)). Thus, they offer a unique opportunity to quantitatively understand the role of mechanics (local and global) in ensuring robust morphogenesis. For that, it is necessary to generate quantitative maps of tissue flows and gene expression, while imposing various mechanical constraints on the gastruloids.

### 16.3. Advances in science and technology to meet challenges

The long-term imaging of 3D multicellular systems such as gastruloids is a challenging task, due to light scattering and phototoxic effects. Multiphoton microscopy enables the imaging of deep (a few 100 *μ*m) tissues, with moderate phototoxicity, and allows fluorescence to be combined with label-free imaging. However, as a point-scanning technique, it is rather slow to image multiple thick specimens in 3D at a minute resolution, which is essential to collect data sets that are representative of specimen shape, size and patterning variability. The advent of light-sheet microscopy provides a means to rapidly image 3D specimens with low phototoxic effects. For instance, the *in toto* imaging of fast-developing embryos such as Drosophila can be achieved with a time resolution on the order of 1 s with subcellular resolution. We expect that the use of multiphoton light-sheet microscopy will be instrumental for the parallel imaging of large scattering specimens at sub-cellular resolution.

To connect collective cell dynamics with cell-scale events, including mechanical behaviours (cell rearrangements, shape changes, division and death) and fluctuations in gene expression, it is necessary to identify and segment individual cells, track them and reconstitute cell lineages. This task is challenging in densely-packed 3D tissues with rapid cellular movements and will greatly benefit from the recent advances in deep learning-based segmentation approaches. Such approaches will also be instrumental in the handling and analysis of high-throughput data sets.

Physical modelling is needed to bridge the cellular and the tissue scales and understand the feedback between 3D cell collective flows and the global geometry of tissue boundaries.

One can envision two complementary theoretical and computational approaches. On one hand, particle-based models are efficient at relating stochastic cellular events to macroscopic tissue behaviour. On the other hand, continuum models that consider tissues as active and complex materials offer a powerful means to identify phases, instabilities, and transitions in gene expression and mechanical behaviour. However, formulating continuum models that couple the dynamics of a 3D active material with the deformations of its boundaries will require the development of new theory and new numerical schemes.

Testing and refining the theoretical predictions of such models will require methods of perturbing the boundary conditions imposed on developing multicellular systems. A promising approach is to embed these systems in synthetic gels whose geometric, mechanical, and chemical properties can be controlled in space and time. This could be achieved by combining the available technology for the light-based mechanical engineering of materials [143] with two-photon chemical patterning [144]. Integrating experimental (imaging, microfabrication, and micropatterning) and theoretical approaches will require the collective effort of physicists, mathematicians, biologists and computational scientists.

### 16.4. Concluding remarks

We expect a mutually beneficial interaction between *in vivo* and *in vitro* systems: *in vivo* systems give an invaluable insight into the evolution of functional structures and gene pathways across species; *in vitro* systems enable the exploration of a wide range of perturbations and experimental conditions, mostly inaccessible *in vivo*. Combining these two approaches is thus an important step to understand morphogenesis from basic principles.

## References

[1] Heemskerk I (2019). Full of potential: pluripotent stem cells for the systems biology of embryonic patterning. Dev. Biol.

[2] Shahbazi MN, Siggia ED and Zernicka-Goetz M 2019 Self-organization of stem cells into embryos: a window on early mammalian development Science 364 948–51 31171690 10.1126/science.aax0164 PMC8300856

[3] Chhabra S, Liu L, Goh R, Kong X and Warmflash A 2019 Dissecting the dynamics of signaling events in the BMP, WNT, and NODAL cascade during self-organized fate patterning in human gastruloids PLoS Biol. 17 e3000498 31613879 10.1371/journal.pbio.3000498 PMC6814242

[4] Britton G, Heemskerk I, Hodge R, Qutub AA and Warmflash A 2019 A novel self-organizing embryonic stem cell system reveals signaling logic underlying the patterning of human ectoderm Development 146 dev179093 31519692 10.1242/dev.179093 PMC6826045

[5] Haremaki T, Metzger JJ, Rito T, Ozair MZ, Etoc F and Brivanlou AH 2019 Self-organizing neuruloids model developmental aspects of Huntington’s disease in the ectodermal compartment Nat. Biotechnol 37 1198–208 31501559 10.1038/s41587-019-0237-5

[6] Xue X (2018). Mechanics-guided embryonic patterning of neuroectoderm tissue from human pluripotent stem cells. Nat. Mater.

[7] Meinhardt H 2008 Models of biological pattern formation: from elementary steps to the organization of embryonic axes Curr. Top. Dev. Biol 81 1–63 18023723 10.1016/S0070-2153(07)81001-5

[8] Maître J-L, Turlier H, Illukkumbura R, Eismann B, Niwayama R, Nédélec F and Hiiragi T 2016 Asymmetric division of contractile domains couples cell positioning and fate specification Nature 536 344–8 27487217 10.1038/nature18958 PMC4998956

[9] Przybyla L, Lakins JN and Weaver VM 2016 Tissue mechanics orchestrate wnt-dependent human embryonic stem cell differentiation Cell Stem Cell 19 462–75 27452175 10.1016/j.stem.2016.06.018 PMC5336327

[10] Brassard JA and Lutolf MP 2019 Engineering stem cell self-organization to build better organoids Cell Stem Cell 24 860–76 31173716 10.1016/j.stem.2019.05.005

[11] Briscoe J and Small S 2015 Morphogen rules: design principles of gradient-mediated embryo patterning Development 142 3996–4009 26628090 10.1242/dev.129452 PMC4712844

[12] Hariharan IK 2015 Organ size control: lessons from Drosophila Dev. Cell 34 255–65 26267393 10.1016/j.devcel.2015.07.012 PMC4547687

[13] Kicheva A, Bollenbach T, Ribeiro A, Valle HP, Lovell-Badge R, Episkopou V and Briscoe J 2014 Coordination of progenitor specification and growth in mouse and chick spinal cord Science 345 1254927 25258086 10.1126/science.1254927 PMC4228193

[14] Sasai N, Kutejova E and Briscoe J 2014 Integration of signals along orthogonal axes of the vertebrate neural tube controls progenitor competence and increases cell diversity PLoS Biol. 12 e1001907 25026549 10.1371/journal.pbio.1001907 PMC4098999

[15] Aguilar-Hidalgo D, Werner S, Wartlick O, González-Gaitán M, Friedrich BM and Jülicher F 2018 Critical point in self-organized tissue growth Phys. Rev. Lett 120 198102 29799239 10.1103/PhysRevLett.120.198102

[16] Shilo BZ and Barkai N 2017 Buffering Global Variability of Morphogen Gradients Dev. Cell 40 429–38 28292422 10.1016/j.devcel.2016.12.012

[17] McDole K, Guignard L, Amat F, Berger A, Malandain G, Royer LA, Turaga SC, Branson K and Keller PJ 2018 In toto imaging and reconstruction of post-implantation mouse development at the single-cell level Cell 175 859–76 30318151 10.1016/j.cell.2018.09.031

[18] Durrieu L, Kirrmaier D, Schneidt T, Kats I, Raghavan S, Hufnagel L, Saunders TE and Knop M 2018 Bicoid gradient formation mechanism and dynamics revealed by protein lifetime analysis Mol. Syst. Biol 14 e8355 30181144 10.15252/msb.20188355 PMC6121778

[19] Sako K 2016 Optogenetic control of nodal signaling reveals a temporal pattern of nodal signaling regulating cell fate specification during gastrulation Cell Rep. 16 866–77 27396324 10.1016/j.celrep.2016.06.036

[20] Guerrero P, Perez-Carrasco R, Zagorski M, Page D, Kicheva A, Briscoe J and Page KM 2019 Neuronal differentiation influences progenitor arrangement in the vertebrate neuroepithelium Development 146 dev176297 31784457 10.1242/dev.176297 PMC6918779

[21] Chabab S, Lescroart F, Rulands S, Mathiah N, Simons BD and Blanpain C 2016 Uncovering the number and clonal dynamics of Mesp1 progenitors during heart morphogenesis Cell Rep. 14 1–10 26725109 10.1016/j.celrep.2015.12.013 PMC4709258

[22] Zhao F, Chen W and Traas J 2018 Mechanical signaling in plant morphogenesis Curr. Opin. Genet. Dev 51 26–30 29727830 10.1016/j.gde.2018.04.001

[23] Bidhendi AJ and Geitmann A 2019 Methods to quantify primary plant cell wall mechanics J. Exp. Bot 70 3615–48 31301141 10.1093/jxb/erz281

[24] Coen E, Kennaway R and Whitewoods C 2017 On genes and form Development 144 4203–13 29183934 10.1242/dev.151910

[25] Braybrook SA and Jönsson H 2016 Shifting foundations: the mechanical cell wall and development Curr. Opin. Plant Biol 29 115–20 26799133 10.1016/j.pbi.2015.12.009

[26] Cosgrove DJ 2018 Nanoscale structure, mechanics and growth of epidermal cell walls Curr. Opin. Plant Biol 46 77–86 30142487 10.1016/j.pbi.2018.07.016

[27] Beauzamy L, Nakayama N and Boudaoud A 2014 Flowers under pressure: ins and outs of turgor regulation in development Ann. Bot 114 1517–33 25288632 10.1093/aob/mcu187 PMC4204789

[28] Hamant O and Haswell ES 2017 Life behind the wall: sensing mechanical cues in plants BMC Biol. 15 59–9 28697754 10.1186/s12915-017-0403-5 PMC5505048

[29] Kollist H, Zandalinas SI, Sengupta S, Nuhkat M, Kangasjärvi J and Mittler R 2019 Rapid responses to abiotic stress: priming the landscape for the signal transduction network Trends Plant Sci. 24 25–37 30401516 10.1016/j.tplants.2018.10.003

[30] Hong L, Dumond M, Zhu M, Tsugawa S, Li C-B, Boudaoud A, Hamant O and Roeder AHK 2018 Heterogeneity and robustness in plant morphogenesis: from cells to organs Annu. Rev. Plant Biol 69 469–95 29505739 10.1146/annurev-arplant-042817-040517

[31] Sigal YM, Zhou R and Zhuang X 2018 Visualizing and discovering cellular structures with super-resolution microscopy Science 361 880–7 30166485 10.1126/science.aau1044 PMC6535400

[32] Turing A 1952 The chemical basis of morphogenesis Phil. Trans. R. Soc. B 237 37–72

[33] Huang A and Saunders TE 2019 A matter of time: formation and interpretation of the Bicoid morphogen gradient Current Topics in Developmental Biology (New York: Academic) 10.1016/bs.ctdb.2019.11.016 32143754

[34] Gregor T, Wieschaus EF, McGregor AP, Bialek W and Tank DW 2007 Stability and nuclear dynamics of the bicoid morphogen gradient Cell 130 141–52 17632061 10.1016/j.cell.2007.05.026 PMC2253672

[35] Kicheva A, Pantazis P, Bollenbach T, Kalaidzidis Y, Bittig T, Jülicher F and González-Gaitán M 2007 Kinetics of morphogen gradient formation Science 315 521–5 17255514 10.1126/science.1135774

[36] Balaskas N, Ribeiro A, Panovska J, Dessaud E, Sasai N, Page KM, Briscoe J and Ribes V 2012 Gene regulatory logic for reading the sonic hedgehog signaling gradient in the vertebrate neural tube Cell 148 273–84 22265416 10.1016/j.cell.2011.10.047 PMC3267043

[37] Wang Y, Wang X, Wohland T and Sampath K 2016 Extracellular interactions and ligand degradation shape the nodal morphogen gradient eLife 5 e13879 27101364 10.7554/eLife.13879 PMC4887204

[38] Roy S, Huang H, Liu S and Kornberg TB 2014 Cytoneme-mediated contact-dependent transport of the Drosophila decapentaplegic signaling protein Science 343 1244624 24385607 10.1126/science.1244624 PMC4336149

[39] Jaeger J 2004 Dynamic control of positional information in the early Drosophila embryo Nature 430 368–71 15254541 10.1038/nature02678

[40] Huang A, Amourda C, Zhang S, Garfield D and Saunders TE 2017 Decoding temporal interpretation of the morphogen bicoid in the early Drosophila embryo eLife 6 262 10.7554/eLife.26258 PMC5515579 28691901

[41] Spratt NT and Haas H 1960 Integrative mechanisms in development of the early chick blastoderm: I. Regulative potentiality of separated parts J. Exp. Zool 145 97–137

[42] Saadaoui M, Rocancourt D, Roussel J, Corson F and Gros J 2020 A tensile ring drives tissue flows to shape the gastrulating amniote embryo Science 367 453–8 31974255 10.1126/science.aaw1965

[43] Engler AJ, Sen S, Sweeney HL and Discher DE 2006 Matrix elasticity directs stem cell lineage specification Cell 126 677–89 16923388 10.1016/j.cell.2006.06.044

[44] Sugimura K, Lenne P-F and Graner F 2016 Measuring forces and stressesin situin living tissues Development 143 186–96 26786209 10.1242/dev.119776

[45] Brunet T (2013). Evolutionary conservation of early mesoderm specification by mechanotransduction in bilateria. Nat. Commun.

[46] Vasiev B, Balter A, Chaplain M, Glazier JA and Weijer CJ 2010 Modeling gastrulation in the chick embryo: formation of the primitive streak PloS One 5 e10571 20485500 10.1371/journal.pone.0010571 PMC2868022

[47] Voiculescu O, Bodenstein L, Lau I-J and Stern CD 2014 Local cell interactions and self-amplifying individual cell ingression drive amniote gastrulation eLife 3 e01817 24850665 10.7554/eLife.01817 PMC4029171

[48] Ståhl PL 2016 Visualization and analysis of gene expression in tissue sections by spatial transcriptomics Science 353 78–82 27365449 10.1126/science.aaf2403

[49] Livshits A, Shani-Zerbib L, Maroudas-Sacks Y, Braun E and Keren K 2017 Structural inheritance of the actin cytoskeletal organization determines the body axis in regenerating hydra Cell Rep. 18 1410–21 28178519 10.1016/j.celrep.2017.01.036

[50] Scimone ML, Cote LE and Reddien PW 2017 Orthogonal muscle fibres have different instructive roles in planarian regeneration Nature 551 623–8 29168507 10.1038/nature24660 PMC6263039

[51] Lardennois A 2019 An actin-based viscoplastic lock ensures progressive body-axis elongation Nature 573 266–70 31462781 10.1038/s41586-019-1509-4 PMC8086821

[52] Cetera M, Juan GRR-S, Oakes PW, Lewellyn L, Fairchild MJ, Tanentzapf G, Gardel ML and Horne-Badovinac S 2014 Epithelial rotation promotes the global alignment of contractile actin bundles during Drosophila egg chamber elongation Nat. Commun 5 5511 25413675 10.1038/ncomms6511 PMC4241503

[53] Huycke TR, Miller BM, Gill HK, Nerurkar NL, Sprinzak D, Mahadevan L and Tabin CJ 2019 Genetic and mechanical regulation of intestinal smooth muscle development Cell 179 90–105 31539501 10.1016/j.cell.2019.08.041 PMC6756183

[54] Livne A, Bouchbinder E and Geiger B 2014 Cell reorientation under cyclic stretching Nat. Commun 5 3938 24875391 10.1038/ncomms4938 PMC4066201

[55] Helm P, Beg MF, Miller MI and Winslow RL 2005 Measuring and mapping cardiac fiber and laminar architecture using diffusion tensor MR imaging Ann. New York Acad. Sci 1047 296–307 16093505 10.1196/annals.1341.026

[56] Braun E and Keren K 2018 Hydraregeneration: closing the loop with mechanical processes in morphogenesis Bioessays 40 1700204 10.1002/bies.201700204 29869336

[57] Maroudas-Sacks Y, Garion L, Shani-Zerbib L, Livshits A, Braun E and Keren K 2020 Topological defects in the nematic order of actin fibres as organization centres of hydra morphogenesis Nat. Phys

[58] Serwane F, Mongera A, Rowghanian P, Kealhofer DA, Lucio AA, Hockenbery ZM and Campàs O 2017 In vivo quantification of spatially varying mechanical properties in developing tissues Nat. Methods 14 181–6 27918540 10.1038/nmeth.4101 PMC5524219

[59] Hannezo E and Heisenberg C-P 2019 Mechanochemical feedback loops in development and disease Cell 178 12–25 31251912 10.1016/j.cell.2019.05.052

[60] Landge AN, Jordan BM, Diego X and Müller P 2020 Pattern formation mechanisms of self-organizing reaction-diffusion systems Dev. Biol 460 2 32008805 10.1016/j.ydbio.2019.10.031 PMC7154499

[61] Diego X, Marcon L, Müller P and Sharpe J 2018 Key features of Turing systems are determined purely by network topology Phys. Rev. X 8 021071

[62] Shyer AE, Huycke TR, Lee C, Mahadevan L and Tabin CJ 2015 Bending gradients: how the intestinal stem cell gets its home Cell 161 569–80 25865482 10.1016/j.cell.2015.03.041 PMC4409931

[63] Guiu J 2019 Tracing the origin of adult intestinal stem cells Nature 570 107–11 31092921 10.1038/s41586-019-1212-5 PMC6986928

[64] Menshykau D, Michos O, Lang C, Conrad L, McMahon AP and Iber D 2019 Image-based modeling of kidney branching morphogenesis reveals GDNF-RET based Turing-type mechanism and pattern-modulating WNT11 feedback Nat. Commun 10 239 30651543 10.1038/s41467-018-08212-8 PMC6484223

[65] Brinkmann F, Mercker M, Richter T and Marciniak-Czochra A 2018 Post-Turing tissue pattern formation: advent of mechanochemistry PloS Comput. Biol 14 e1006259 29969460 10.1371/journal.pcbi.1006259 PMC6047832

[66] Recho P, Hallou A and Hannezo E 2019 Theory of mechanochemical patterning in biphasic biological tissues Proc. Natl Acad. Sci. USA 116 5344–9 30819884 10.1073/pnas.1813255116 PMC6431232

[67] Boocock D, Hino N, Ruzickova N, Hirashima T and Hannezo E 2021 Theory of mechano-chemical patterning and optimal migration in cell monolayers Nat. Phys 17 267–74

[68] Beccari L, Moris N, Girgin M, Turner DA, Baillie-Johnson P, Cossy A-C, Lutolf MP, Duboule D and Arias AM 2018 Multi-axial self-organization properties of mouse embryonic stem cells into gastruloids Nature 562 272–6 30283134 10.1038/s41586-018-0578-0

[69] Eiraku M, Takata N, Ishibashi H, Kawada M, Sakakura E, Okuda S, Sekiguchi K, Adachi T and Sasai Y 2011 Self-organizing optic-cup morphogenesis in three-dimensional culture Nature 472 51–6 21475194 10.1038/nature09941

[70] Sasai Y 2013 Cytosystems dynamics in self-organization of tissue architecture Nature 493 318–26 23325214 10.1038/nature11859

[71] Theraulaz G and Bonabeau E 1999 A brief history of stigmergy Artif. Life 5 1–20 10633572 10.1162/106454699568700

[72] Cerchiari AE 2015 A strategy for tissue self-organization that is robust to cellular heterogeneity and plasticity Proc. Natl Acad. Sci. USA 112 2287–92 25633040 10.1073/pnas.1410776112 PMC4343104

[73] Marchetti MC, Joanny JF, Ramaswamy S, Liverpool TB, Prost J, Rao M and Simha RA 2013 Hydrodynamics of soft active matter Rev. Mod. Phys 85 1143

[74] Buzi G, Lander AD and Khammash M 2015 Cell lineage branching as a strategy for proliferative control BMC Biol. 13 13 25857410 10.1186/s12915-015-0122-8 PMC4378012

[75] Eng C-HL 2019 Transcriptome-scale super-resolved imaging in tissues by RNA seqFISH+ Nature 568 235–9 30911168 10.1038/s41586-019-1049-y PMC6544023

[76] Angelo M 2014 Multiplexed ion beam imaging of human breast tumors Nat. Med 20 436–42 24584119 10.1038/nm.3488 PMC4110905

[77] Nerurkar NL, Mahadevan L and Tabin CJ 2017 BMP signaling controls buckling forces to modulate looping morphogenesis of the gut Proc. Natl Acad. Sci. USA 114 2277–82 28193855 10.1073/pnas.1700307114 PMC5338480

[78] Toda S, Blauch LR, Tang SKY, Morsut L and Lim WA 2018 Programming self-organizing multicellular structures with synthetic cell–cell signaling Science 361 eaat0271 10.1126/science.aat0271 PMC6492944 29853554

[79] Choi J-M, Holehouse AS and Pappu RV 2020 Physical principles underlying the complex biology of intracellular phase transitions Annu. Rev. Biophys 49 107–33 32004090 10.1146/annurev-biophys-121219-081629 PMC10715172

[80] Delarue M 2018 mTORC1 controls phase separation and the biophysical properties of the cytoplasm by tuning crowding Cell 174 338–49 29937223 10.1016/j.cell.2018.05.042 PMC10080728

[81] Hyman AA, Weber CA and Jülicher F 2014 Liquid–liquid phase separation in biology Annu. Rev. Cell Dev. Biol 30 39–58 25288112 10.1146/annurev-cellbio-100913-013325

[82] Langdon EM and Gladfelter AS 2018 A new lens for RNA localization: liquid–liquid phase separation Annu. Rev. Microbiol 72 255–71 30200855 10.1146/annurev-micro-090817-062814

[83] Langdon EM (2018). mRNA structure determines specificity of a polyQ-driven phase separation. Science.

[84] McSwiggen DT, Mir M, Darzacq X and Tjian R 2019 Evaluating phase separation in live cells: diagnosis, caveats, and functional consequences Genes Dev. 33 1619–34 31594803 10.1101/gad.331520.119 PMC6942051

[85] Shin Y, Berry J, Pannucci N, Haataja MP, Toettcher JE and Brangwynne CP 2017 Spatiotemporal control of intracellular phase transitions using light-activated optoDroplets Cell 168 159–71 28041848 10.1016/j.cell.2016.11.054 PMC5562165

[86] Smola MJ, Rice GM, Busan S, Siegfried NA and Weeks KM 2015 Selective 2′-hydroxyl acylation analyzed by primer extension and mutational profiling (SHAPE-MaP) for direct, versatile and accurate RNA structure analysis Nat. Protocols 10 1643–69 26426499 10.1038/nprot.2015.103 PMC4900152

[87] Wang C, Han B, Zhou R and Zhuang X 2016 Real-time imaging of translation on single mRNA transcripts in live cells Cell 165 990–1001 27153499 10.1016/j.cell.2016.04.040 PMC4905760

[88] Wei M-T, Elbaum-Garfinkle S, Holehouse AS, Chen CC-H, Feric M, Arnold CB, Priestley RD, Pappu RV and Brangwynne CP 2017 Phase behaviour of disordered proteins underlying low density and high permeability of liquid organelles Nat. Chem 9 1118–25 29064502 10.1038/nchem.2803 PMC9719604

[89] Nüsslein-Volhard C and Wieschaus E 1980 Mutations affecting segment number and polarity in Drosophila Nature 287 795–801 6776413 10.1038/287795a0

[90] Takahashi K and Yamanaka S 2006 Induction of pluripotent stem cells from mouse embryonic and adult fibroblast cultures by defined factors Cell 126 663–76 16904174 10.1016/j.cell.2006.07.024

[91] Materna SC and Davidson EH 2007 Logic of gene regulatory networks Curr. Opin. Biotechnol 18 351–4 17689240 10.1016/j.copbio.2007.07.008 PMC2031216

[92] Wolpert L 1969 Positional information and the spatial pattern of cellular differentiation J. Theor. Biol 25 1–47 4390734 10.1016/s0022-5193(69)80016-0

[93] Yang Y 1997 Relationship between dose, distance and time in sonic hedgehog-mediated regulation of anteroposterior polarity in the chick limb Development 124 4393–404 9334287 10.1242/dev.124.21.4393

[94] Warburton D (2005). Molecular mechanisms of early lung specification and branching morphogenesis. Pediatr. Res.

[95] Sick S, Reinker S, Timmer J and Schlake T 2006 WNT and DKK determine hair follicle spacing through a reaction–diffusion mechanism Science 314 1447–50 17082421 10.1126/science.1130088

[96] Shyer AE, Rodrigues AR, Schroeder GG, Kassianidou E, Kumar S and Harland RM 2017 Emergent cellular self-organization and mechanosensation initiate follicle pattern in the avian skin Science 357 811–5 28705989 10.1126/science.aai7868 PMC5605277

[97] Oster GF, Murray JD and Harris AK 1983 Mechanical aspects of mesenchymal morphogenesis J. Embryol. Exp. Morphol 83 125 6663234

[98] Minc N and Piel M 2012 Predicting division plane position and orientation Trends Cell Biol. 22 193–200 22321291 10.1016/j.tcb.2012.01.003

[99] Hashimoto H and Munro E 2018 Dynamic interplay of cell fate, polarity and force generation in ascidian embryos Curr. Opin. Genet. Dev 51 67–77 30007244 10.1016/j.gde.2018.06.013 PMC6543843

[100] Cheng X and Ferrell JE 2019 Spontaneous emergence of cell-like organization in Xenopus egg extracts Science 366 631–7 31672897 10.1126/science.aav7793 PMC7839252

[101] Minc N, Burgess D and Chang F 2011 Influence of cell geometry on division-plane positioning Cell 144 414–26 21295701 10.1016/j.cell.2011.01.016 PMC3048034

[102] Wühr M, Tan ES, Parker SK, Detrich HW 3rd and Mitchison TJ 2010 A model for cleavage plane determination in early amphibian and fish embryos Curr. Biol 20 2040–5 21055946 10.1016/j.cub.2010.10.024 PMC3031131

[103] Pierre A, Sallé J, Wühr M and Mine N 2016 Generic theoretical models to predict division patterns of cleaving embryos Dev. Cell 39 667–82 27997824 10.1016/j.devcel.2016.11.018 PMC5180451

[104] Mitchison T, Wühr M, Nguyen P, Ishihara K, Groen A and Field CM 2012 Growth, interaction, and positioning of microtubule asters in extremely large vertebrate embryo cells Cytoskeleton 69 738–50 22786885 10.1002/cm.21050 PMC3690567

[105] Kimura K and Kimura A 2011 Intracellular organelles mediate cytoplasmic pulling force for centrosome centration in the Caenorhabditis elegans early embryo Proc. Natl Acad. Sci. USA 108 137–42 21173218 10.1073/pnas.1013275108 PMC3017145

[106] Sallé J, Xie J, Ershov D, Lacassin M, Dmitrieff S and Minc N 2019 Asymmetric division through a reduction of microtubule centering forces J. Cell Biol 218 771–82 30563876 10.1083/jcb.201807102 PMC6400563

[107] Xiong F 2014 Interplay of cell shape and division orientation promotes robust morphogenesis of developing epithelia Cell 159 415–27 25303534 10.1016/j.cell.2014.09.007 PMC4273647

[108] Maître J-L, Niwayama R, Turlier H, Nédélec F and Hiiragi T 2015 Pulsatile cell-autonomous contractility drives compaction in the mouse embryo Nat. Cell Biol 17 849–55 26075357 10.1038/ncb3185

[109] White MD, Álvarez YD, Fierro-González JC, Bissiere S and Plachta N 2015 Cortical tension allocates the first inner cells of the mammalian embryo Dev. Cell 34 435–47 26279486 10.1016/j.devcel.2015.07.004

[110] Leonavicius K, Royer C, Preece C, Davies B, Biggins JS and Srinivas S 2018 Mechanics of mouse blastocyst hatching revealed by a hydrogel-based microdeformation assay Proc. Natl Acad. Sci. USA 115 10375–80 30232257 10.1073/pnas.1719930115 PMC6187134

[111] Dumortier JG, Le Verge-Serandour M, Tortorelli AF, Mielke A, de Plater L, Turlier H and Maître J-L 2019 Hydraulic fracturing and active coarsening position the lumen of the mouse blastocyst Science 365 465–8 31371608 10.1126/science.aaw7709

[112] Chan CJ, Costanzo M, Ruiz-Herrero T, Mönke G, Petrie RJ, Bergert M, Diz-Muñoz A, Mahadevan L and Hiiragi T 2019 Hydraulic control of mammalian embryo size and cell fate Nature 571 112–6 31189957 10.1038/s41586-019-1309-x

[113] Zenker J, White MD, Gasnier M, Álvarez YD, Lim HYG, Bissiere S, Biro M and Plachta N 2018 Expanding actin rings zipper the mouse embryo for blastocyst formation Cell 173 776 29576449 10.1016/j.cell.2018.02.035

[114] Fierro-González JC, White MD, Silva JC and Plachta N 2013 Cadherin-dependent filopodia control preimplantation embryo compaction Nat. Cell Biol 15 1424 24270889 10.1038/ncb2875

[115] Wu P-H (2018). A comparison of methods to assess cell mechanical properties. Nat. Methods.

[116] O’Farrell PH (2015). Growing an embryo from a single cell: a hurdle in animal life. Cold Spring Harb. Perspect. Biol.

[117] Ferree PL, Deneke VE and Di Talia S 2016 Measuring time during early embryonic development Semin. Cell Dev. Biol 55 80–8 26994526 10.1016/j.semcdb.2016.03.013 PMC4903905

[118] Deneke VE, Melbinger A, Vergassola M and Di Talia S 2016 Waves of Cdk1 activity in S phase synchronize the cell cycle in Drosophila embryos Dev. Cell 38 399–412 27554859 10.1016/j.devcel.2016.07.023 PMC4999262

[119] Vergassola M, Deneke VE and Di Talia S 2018 Mitotic waves in the early embryogenesis of Drosophila: bistability traded for speed Proc. Natl Acad. Sci. USA 115 E2165–74 29449348 10.1073/pnas.1714873115 PMC5878005

[120] Deneke VE, Puliafito A, Krueger D, Narla AV, De Simone A, Primo L, Vergassola M, De Renzis S and Di Talia S 2019 Self-organized nuclear positioning synchronizes the cell cycle in Drosophila embryos Cell 177 925–41 30982601 10.1016/j.cell.2019.03.007 PMC6499673

[121] Shamipour S, Kardos R, Xue SL, Hof B, Hannezo E and Heisenberg CP 2019 Bulk actin dynamics drive phase segregation in zebrafish oocytes Cell 177 1463–79 31080065 10.1016/j.cell.2019.04.030

[122] Field CM, Wühr M, Anderson GA, Kueh HY, Strickland D and Mitchison TJ 2011 Actin behavior in bulk cytoplasm is cell cycle regulated in early vertebrate embryos J. Cell Sci 124 2086–95 21610091 10.1242/jcs.082263 PMC3104037

[123] Mogilner A and Manhart A 2017 Intracellular fluid mechanics: coupling cytoplasmic flow with active cytoskeletal gel Annu. Rev. Fluid Mech 50 347–70

[124] Deneke VE and Di Talia S 2018 Chemical waves in cell and developmental biology J. Cell Biol 217 1193–204 29317529 10.1083/jcb.201701158 PMC5881492

[125] Mittasch M 2018 Non-invasive perturbations of intracellular flow reveal physical principles of cell organization Nat. Cell Biol 20 344–51 29403036 10.1038/s41556-017-0032-9

[126] Levayer R, Hauert B and Moreno E 2015 Cell mixing induced by myc is required for competitive tissue invasion and destruction Nature 524 476–80 26287461 10.1038/nature14684

[127] Shaya O 2017 Cell–cell contact area affects Notch signalling and Notch-dependent patterning Dev. Cell 40 505–11 28292428 10.1016/j.devcel.2017.02.009 PMC5435110

[128] Guisoni N, Martinez-Corral R, Garcia-Ojalvo J and de Navascués J 2017 Diversity of fate outcomes in cell pairs under lateral inhibition Development 144 1177–86 28174242 10.1242/dev.137950

[129] Hunter G, Hadjivasiliou L, Bonin H, He L, Perrimon N, Charras G and Baum B 2016 Coordinated control of Notch/Delta signalling and cell cycle progression drives lateral inhibition-mediated tissue patterning Development 143 2305–10 27226324 10.1242/dev.134213 PMC4958321

[130] Trylinski M, Mazouni K and Schweisguth F 2017 Intra-lineage fate decisions involve activation of Notch receptors basal to the midbody in Drosophila sensory organ precursor cells Curr. Biol 27 2239–47 28736165 10.1016/j.cub.2017.06.030

[131] Falo-Sanjuan J, Lammers NC, Garcia HG and Bray SJ 2019 Enhancer priming enables fast and sustained transcriptional responses to Notch signalling Dev. Cell 50 411–25 31378591 10.1016/j.devcel.2019.07.002 PMC6706658

[132] Khait I, Orsher Y, Golan O, Binshtok U, Gordon-Bar N, Amir-Zilberstein L and Sprinzak D 2016 Quantitative analysis of Delta-like 1 membrane dynamics elucidates the role of contact geometry on Notch signalling Cell Rep. 14 225–33 26748704 10.1016/j.celrep.2015.12.040

[133] Trylinski M and Schweisguth F 2019 Activation of Arp2/3 by WASp is essential for the endocytosis of Delta only during cytokinesis in Drosophila Cell Rep. 28 1–10 31269431 10.1016/j.celrep.2019.06.012

[134] Barone V, Lang M, Krens SG, Pradhan SJ, Shamipour S, Sako K, Sikora M, Guet CC and Heisenberg C-P 2017 An effective feedback loop between cell–cell contact duration and morphogen signalling determines cell fate Dev. Cell 43 198–211 29033362 10.1016/j.devcel.2017.09.014

[135] Viswanathan R, Necakov A, Trylinski M, Harish RK, Krueger D, Esposito E, Schweisguth F, Neveu P and De Renzis S 2019 Optogenetic inhibition of Delta reveals digital Notch signalling output during tissue differentiation EMBO Rep. 20 e47999 31668010 10.15252/embr.201947999 PMC6893285

[136] Collinet C, Rauzi M and Lenne P-F 2015 Lecuit T local and tissue-scale forces drive oriented junction growth during tissue extension Nat. Cell Biol 17 1247–58 26389664 10.1038/ncb3226

[137] Lye CM, Blanchard GB, Naylor HW, Muresan L, Huisken J, Adams RJ and Sanson B 2015 Mechanical coupling between endoderm invagination and axis extension in Drosophila PloS Biol. 13 e1002292 26544693 10.1371/journal.pbio.1002292 PMC4636290

[138] Dicko M, Saramito P, Blanchard GB, Lye CM, Sanson B and Étienne J 2017 Geometry can provide long-range mechanical guidance for embryogenesis PloS Comput. Biol e1005443 28346461 10.1371/journal.pcbi.1005443 PMC5386319

[139] Streichan SJ, Lefebvre MF, Noll N, Wieschaus EF and Shraiman BI 2018 Global morphogenetic flow is accurately predicted by the spatial distribution of myosin motors eLife 7 27454 10.7554/eLife.27454 PMC5843464 29424685

[140] de Medeiros G (2020). Cell and tissue manipulation with ultrashort infrared laser pulses in light-sheet microscopy. Sci. Rep.

[141] Sugimura K, Lenne P-F and Graner F 2016 Measuring forces and stresses in situ in living tissues Development 143 186–96 26786209 10.1242/dev.119776

[142] Beccari L, Moris N, Girgin M, Turner DA, Baillie-Johnson P, Cossy A-C, Lutolf MP, Duboule D and Arias AM 2018 Multi-axial self-organization properties of mouse embryonic stem cells into gastruloids Nature 562 272–6 30283134 10.1038/s41586-018-0578-0

[143] Pasturel A, Strale P-O and Studer V 2020 Tailoring common hydrogels into 3D cell culture templates. Adv. Healthcare Mater 9 2000519 10.1002/adhm.202000519 32743980

[144] Broguiere N, Lüchtefeld I, Traschel L, Mazunin D, Bode J, Lutolf MP and Zenobi-Wong M 2019 Morphogenesis guided by 3D patterning of growth factors in biological matrices Adv. Mater 32 1908299 10.1002/adma.201908299 32390195

